# The Halogen Bond to Ethers - Prototypic Molecules and Experimental Electron Density

**DOI:** 10.1021/acsomega.4c05124

**Published:** 2024-08-05

**Authors:** Annika Schmidt, Anna Krupp, Johannes Kleinheider, Tamara M. L. Binnenbrinkmann, Ruimin Wang, Ulli Englert, Carsten Strohmann

**Affiliations:** † Inorganic Chemistry, TU Dortmund University, Otto-Hahn-Straße 6, 44227 Dortmund, Germany; ‡ Institute of Inorganic Chemistry, RWTH Aachen University, Landoltweg 1, 52056 Aachen, Germany; § Institute of Molecular Science, Key Laboratory of Chemical Biology and Molecular Engineering of the Education Ministry, Shanxi University, Taiyuan, Shanxi 030006, China

## Abstract

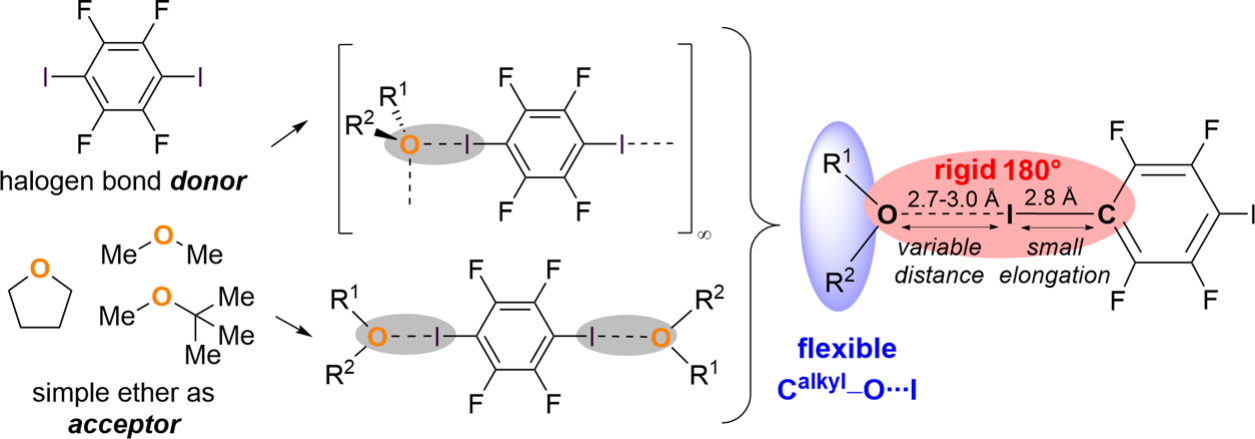

Halogen bonds to dialkyl ether molecules have remained largely unexplored. We here address the synthesis and the structural chemistry of the first halogen-bonded noncyclic alkyl ethers, combining 1,4-diiodotetrafluorobenzene and the prototypic or commonly used ethers dimethyl ether, tetrahydrofuran, and methyl-*tert*-butyl ether as halogen acceptors. Two different structural motifs based on moderately strong halogen bonds were obtained: Discrete trimolecular aggregates are formed, and unexpected halogen-bonded supramolecular chain adducts feature oxygen-bifurcated halogen bonds with 1:1 donor:acceptor ratio. Both structure types may be selectively obtained even for the same ether by adjusting the stoichiometry in the crystallization experiments. The geometric features of the etheric oxygen center were found to be flexible, in contrast to the almost linear geometry about the halogen donor atom. A high-resolution X-ray diffraction experiment on the extended adduct of dimethyl ether allowed us to study the electronic details of the acceptor-bifurcated I···O···I halogen bonds. The electron density in the bond critical points and derived properties such as the Laplacian indicate essentially electrostatic interactions and explain the geometrical flexibility of ethers in halogen bonds. Our studies demonstrate the great versatility of ethers as halogen bond acceptors, that can occur in many geometrical arrangements and whose contribution to nature’s structural designs should not be underestimated.

## Introduction

Classically, halogen atoms in halo-organyls are characterized as electronegative regions. However, a large amount of structures possess an attractive interaction of these electronegative regions with further electronegative units, like Lewis-bases.^1,2^ This apparently paradoxical behavior can be attributed to an inhomogeneous electron density distribution about the halogen atom: the so-called σ-hole.^3,4^ The formation of this σ-hole is dependent on the polarizability of the halogen atom and therefore increases within the order X = F, Cl, Br to I.^4^ Likewise, the attractive interaction with Lewis-basic units like N- or O atoms, known as halogen bond, also increases within this order.^5^ Typically, the bond distance between the halogen bond donor (i.e., the halogen atom X of halo-organyls) and the halogen bond acceptor (i.e., the Lewis-basic atom LB) is noticeably smaller than the sum of their van der Waals radii. Halogen bonds are highly directional,^1,2b^ with comparatively rigid LB–X–C bond angles closer to 180° than in the case of hydrogen bonds. These geometric features have been consistently reproduced in experimental and theoretical studies. Different explanations concerning the nature of halogen bonds have been discussed in literature, including electrostatic interactions, charge-transfer interactions, dispersion and polarization effects.^1,2,6^

Next to their study and comprehension, halogen bonds are also used, for example in biological systems. The synergy of several noncovalent intermolecular halogen bond interactions between N- and O atoms from amino acids or sugars to halogen atoms can be used to build biological 3D-features.^7^ Notably, thyroid hormones are a class of naturally halogenated hormones. They include halogen I···O bonds that play an important role in the recognition process.^8^ Also, in the field of pharmaceuticals, halogen-substitution, especially with higher halogen homologues, is an upcoming field of research interest,^1^ rendering the exact geometric features of halogen bonds and their prediction essential for targeted drug design techniques.

A plethora of N···X halogen bonds are known and well-studied in literature,^9−11^ but halogen bonds to oxygen atoms and in particular to sp^3^-oxygen atoms in ethers are less investigated, presumably due to their increased steric hindrance in comparison to sp^2^-carbonyl oxygen.^12^

The earliest example of a crystallographically investigated halogen bond with an ether acceptor molecule is the study of the structure of 1,4-dioxane and elemental bromine by Hassel et al. from 1954.^13^ Since these early analyses, further systems with etheric oxygen acceptors have been found, but many of them are characterized by additional noncovalent intermolecular interactions, like hydrogen bonds, π–π-stacking interactions^14^ or N···X-halogen bonds.^14,15^ Also bivalent etheric halogen bond acceptors like dioxane have been presented.^16^

Even though these solid-state structures prove the importance of halogen bonds in the network of intermolecular (weak) interactions once again, a targeted study requires prototypic molecular systems preferably uncontaminated by additional close contacts. However, these simple systems naturally are the most unstable and hence the most challenging ones.

Popular ethers, like tetrahydrofuran or diethyl ether, play an important role as solvents for many organic syntheses. Therefore, their interaction with halogen atoms may have an impact on the reactivity of many organic processes involving halo-organyls.

**Figure 1:**
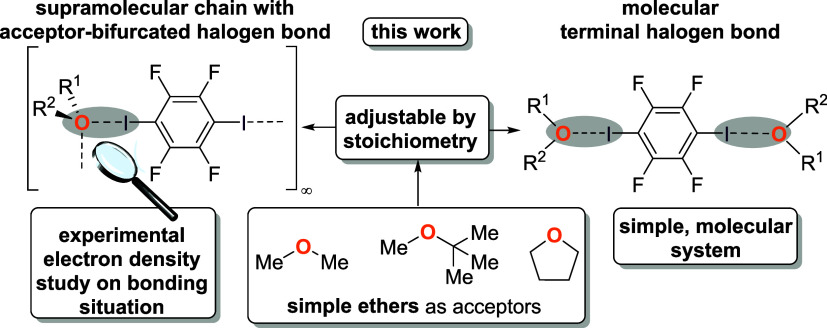
Simple ethers were used as halogen bond acceptors and show two structural motifs (supramolecular chain with acceptor-bifurcated halogen bond vs. molecular terminal halogen bond) that are adjustable by stoichiometry. Experimental electron density studies help to explain the bonding situation.

Herein, we present molecular halogen bond aggregates with methyl-*tert*-butyl ether, tetrahydrofuran and dimethyl ether (Figure 1). Two different halogen bond motifs were obtained, and the equilibrium in between the supramolecular chain and molecular aggregate can be influenced by the stoichiometry of these frequently used solvent molecules. As only halogen bond interactions and no further short intermolecular interactions are observed, such systems are particularly suitable to gain insight into the bonding situation of ether O···I halogen bonds by experimental electron density studies based on high resolution single crystal X-ray diffraction. These insights allow to explain the geometric flexibility of the O_sp^3^_···I halogen bond interaction: Our experiments can address discrete molecular and extended structures with the same tools whereas theory might well recur to different approaches.

## Experimental Section

In the absence of oxygen and water, 1,4-diiodotetrafluorobenzene and the ethers mtbe, thf and dme were dissolved in predried *n*-pentane. Compounds **1**-**5** were obtained as crystals suitable for X-ray diffraction after crystallization at −80 °C. Experimental details and further data on refinements can be found in the Supporting Information (SI). Atomic coordinates and other structural parameters of **1**–**5**, have been deposited with the Cambridge Crystallographic Data Centre (CCDC numbers 2334578 (for **1**), 2334577 (for **2**), 2334576 (for **3**), 2334579 (for the independent atom model for **4**), 2334580 (for the multipole model for **4**), and 2334581 (for **5**)).

## Results and Discussion

By cocrystallization of 1,4-diiodotetrafluorobenzene with the ether partners methyl-*tert*-butyl ether (mtbe), tetrahydrofuran (thf) and dimethyl ether (dme) in different ratios (i.e., 1:1 and 2:1) at −80 °C, highly temperature-sensitive single-crystals suitable for single crystal X-ray diffraction were obtained.

These simple ethers, which are commonly used as solvents in broad fields of chemistry or which are prototypic for applications like quantum chemical calculations, form two distinct structural motifs in halogen bonds to 1,4-diiodotetrafluorobenzene. Interestingly, thf and dimethyl ether can form both motifs in dependency of the used stoichiometric amount of the ether. Figure 1 depicts both motifs in a schematic fashion. A supramolecular acceptor-bifurcated halogen bond forms an extended solid state structure whereas the halogen bond between a single XB donor and acceptor results in a discrete molecular aggregate.

**Figure 2:**
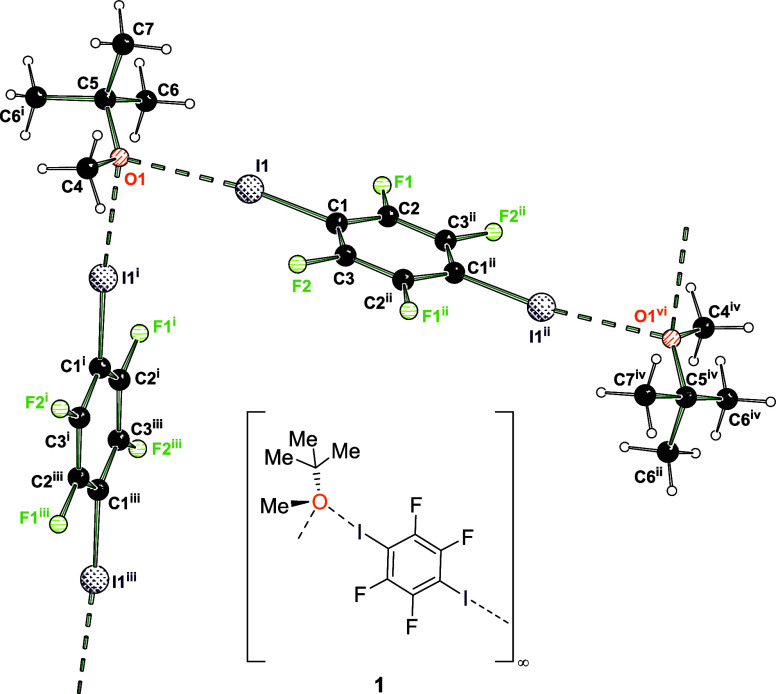
Solid-state structure of halogen bond adduct formed by 1,4-diiodotetrafluorobenzene and mtbe (**1**). Selected distances [Å] and angles [°]: I1···O1 2.9699(9), C1–I1 2.0847(11), C1–I1···O1 172.44(4), I1···O1···I1^i^ 91.61(3); angle between normal on plane A(O1,C4,C5) and bond C1–I1 50.7; i = *x*, 0.5–*y*, *z*; ii = 1–*x*, 1–*y*, – *z*, iii = 1–*x*, – 0.5+*y*, −*z*; iv = 1–*x*, 0.5+*y*, −*z*.

Figure 2 shows the solid-state structure of the presented halogen bond adduct with mtbe (**1**). **1** is a halogen-bonded supramolecular chain based on a structure-forming halogen bond interaction between I1 as halogen bond donor and O1 as halogen bond acceptor. The oxygen center acts as a bifurcated acceptor. We note that bifurcated halogen bonds are mostly associated with two (or more) XB acceptors per XB donor site,^17^ but the idea of acceptor-bifurcation has also been recognized.^18^

This structural motif is known for cases in which oxygen atoms, as halogen bond acceptors, are more pronounced lone pair donors, such as in alcohols,^19^ N–O-compounds,^20^ sulfoxides,^21^ phosphine oxides^22^ and carbonyls.^23^ According to a search in the CSD database,^24^ this behavior has so far not been reported for structures of etheric halogen bonds with iodine as the halogen bond donor.

In **1**, the I1···O1 bond length is 2.9699(9) Å and the contraction relative to the sum of van der Waals radii is 16%.^25^ The I1···O1···I1^i^ angle is 91.61(3)°. Formation of the halogen bond results in an only slightly elongated covalent C–I bond of 2.0847(11) Å. In comparison, the C–I bond length in the starting material 1,4-diodotetrafluorobenzene is 2.0737(6) Å whereas it is more strongly elongated in the nitrogen halogen bond adduct with quinuclidine to 2.1153(6) Å.^9^

**Figure 3:**
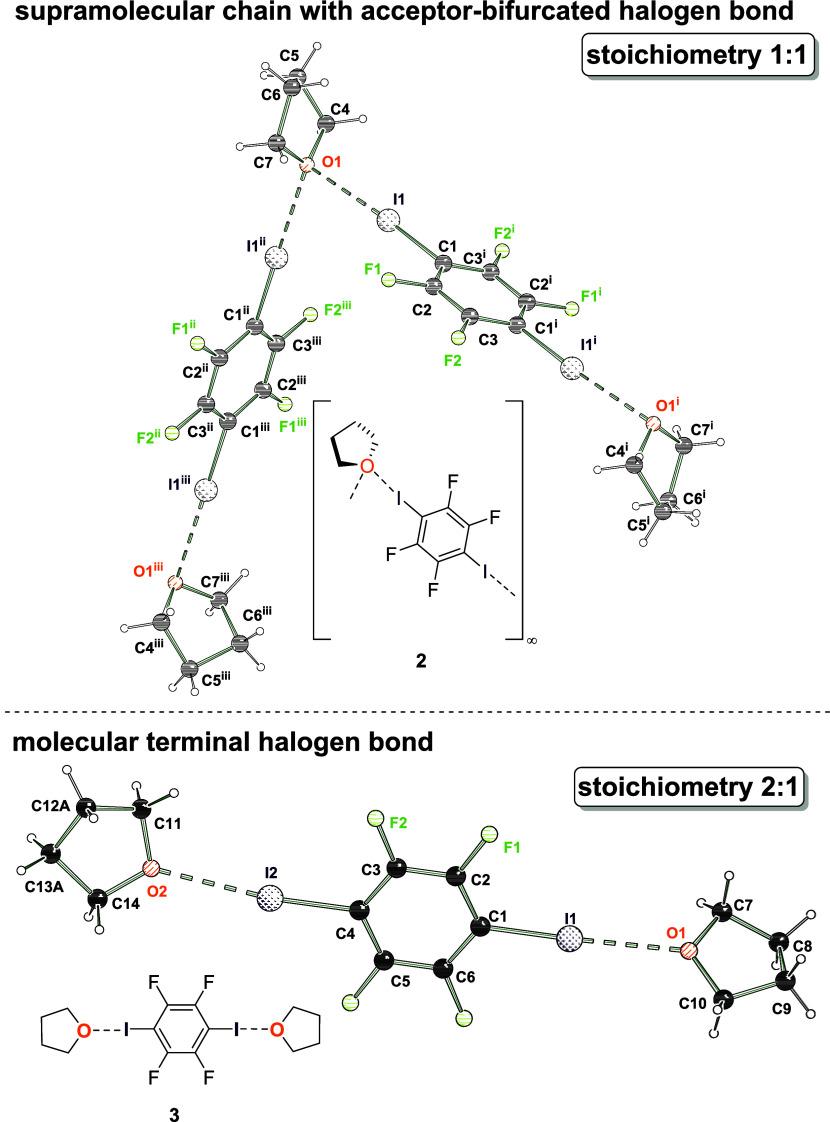
Solid-state structures of halogen bond adducts formed by 1,4-diiodotetrafluorobenzene and thf. Supramolecular chain with acceptor-bifurcated halogen bond motif (**2**, top) and molecular halogen bond motif (**3**, bottom). Selected distances [Å] and angles [°] for **2**: I1···O1 2.9035(13), I1–C1 2.0842(16), C1–I1···O1 176.41(6), I1^i^···O1···I1 89.44(5); angle of normal of area A(O1,C4,C7) and bond C1–I1 36.9; i = 1–*x*,1–*y*,1–*z*; ii = x, 0.5+*y*, *z*; iii = 1–*x*, – 0.5+*y*, 1–*z*. Selected bond lengths [Å] and angles [°] for **3**: I1···O1 2.785(2), C1–I1 2.086(2), I2···O2 2.841(2), C4–I2 2.088(3), C1–I1···O1 177.09(9), C4–I2···O2 173.47(9); angle between normal on plane A(O1,C7,C10) and bond C1–I1 76.1 (large deviation from 90° due to disorder).

Interestingly, by using thf as etheric acceptor, depending on the ratio of the bond partners, two varying structural motifs were obtained: again a halogen-bonded supramolecular chain based on oxygen-bifurcated halogen bonds (Figure 3, top) and a discrete trimolecular aggregate featuring unbranched halogen bonds (Figure 3, bottom). We have not been able to obtain the latter motif with mtbe even after variation of the stoichiometric donor–acceptor ratio.

In **2**, the I1···O1 distance is 2.9035(13) Å (18% contraction relative to the sum of the van der Waals radii)^25^ and slightly shorter than in the analogous halogen bond with mtbe. The C–I bond distance amounts to 2.0842(16) Å, and the bifurcating angle at the XB acceptor site I1^i^···O1···I1 is 89.44(5)° and thus slightly smaller than in **1**. This solid-state structure displays a second example for a bifurcating ether oxygen center in a halogen bond.

However, by adjusting the stoichiometric ratio to a 2:1 excess of thf, a trimolecular aggregate with unbranched XBs was observed (**3**). Herein, the I···O bond distances are 2.785(2) Å for I1–O1 and 2.841(2) Å for I2···O2 and show a contraction relative to the sum of van der Waals radii of 21% and 20%, respectively.^25^ The I···O distances are significantly shorter due to the single-accepting character of the oxygen center of thf in comparison to **2**. However, the C–I bond distances are not significantly elongated in contrast to structures **1** and **2** with values of 2.086(2) Å and 2.088(3) Å.

Importantly, it can be stated that the same halogen bond acceptor is able to show both structural motifs (oxygen-bifurcated, supramolecular bifurcated halogen bond vs. unbranched molecular halogen bond) depending on the ratio of donor to acceptor molecule in the synthesis of the crystals.

**Figure 4:**
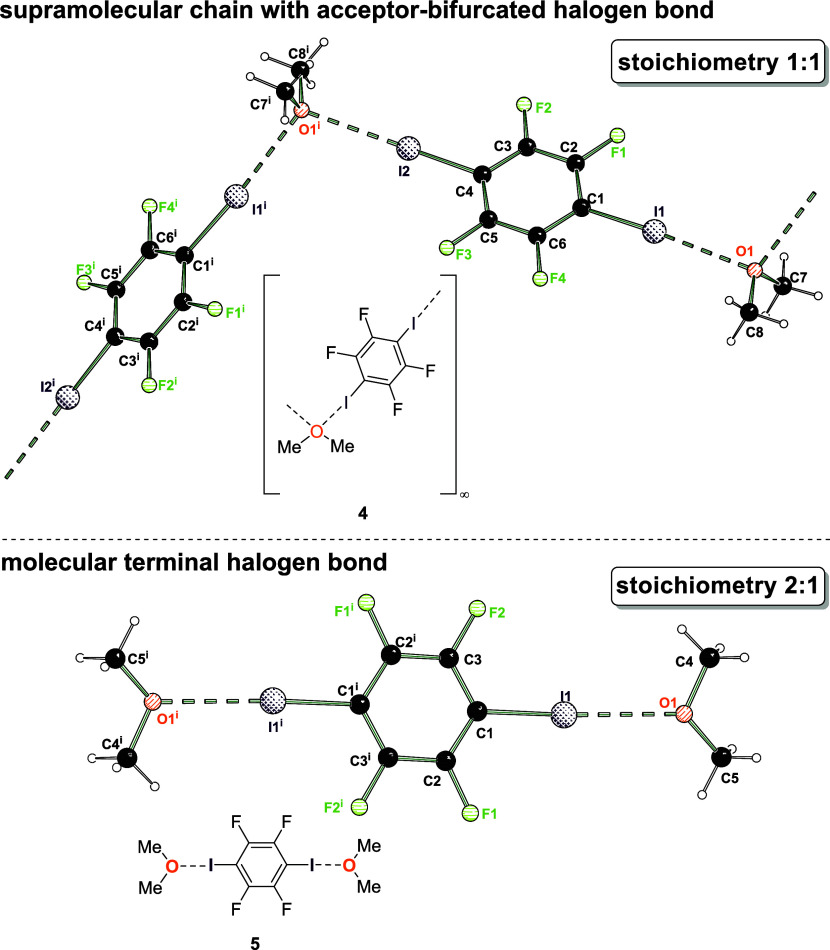
Solid-state structures of halogen bond adducts formed by 1,4-diiodotetrafluorobenzene and dme. Supramolecular chain with acceptor-bifurcated halogen bond motif (**4**, top) and molecular halogen bond motif (**5**, bottom). Selected distances [Å] and angles [°] for **4** (AIM refinement): I2···O1 2.8840(7), C1–I1 2.0814(8), I1···O1^i^ 2.932(6), C4–I2 2.0836(8), C4–I2···O1 175.65(2), C1–I1···O1^i^ 170.77(2), I1···O1^i^···I2^i^ 108.10(2); angle of normal of area A(O1,C7,C8) and bond C4–I2 33.29; i = 1–*x*, 0.5+*y*, 1.5–*z*. Selected bond lengths [Å] and angles [°] for **5**: I1–O1 2.846(3), C1–I1 2.082(3), C1–I1···O1 175.85(12); angle between normal on plane A(O1,C4,C5) and bond C1–I1 84.5; i = 1–*x*, 1–*y*, −*z*.

This behavior was further analyzed for the smallest possible ether, dimethyl ether (Figure 4). This ether is exceptionally present in model-systems for halogen bonds in the context of quantum chemical calculations.^26^ The ability of dimethyl ether to form even oxygen-bifurcated halogen bonds to strong Cl-, Br- and I-donors has been investigated by Raman and FTIR spectroscopy and confirmed in liquid noble gases, but no single crystals were isolable.^27^ In our study, we are now able to present the solid-state structures of halogen bonds with dimethyl ether and 1,4-diiodotetrafluorobenzene.

Again, in dependency of the used stoichiometric amounts of 1,4-diiodotetrafluorobenzene and dme, two structural motifs were obtained that are characterized by a supramolecular bifurcated vs. a molecular halogen bond, analogous to the situation with thf. With all three etheric halogen bond acceptors the so far rare structural motif of oxygen-bifurcated halogen bonds was obtained, proving that this interaction should not be neglected.

The halogen-bonded supramolecular chain halogen bond **4** shows I–O bond distances of 2.8832(5) Å and 2.9304(5) Å and a contraction relative to the sum of van der Waals radii of 19% and 17%, respectively.^25^ The obtained halogen bond lengths of the oxygen-bifurcated systems in all structures do vary to some extent but are still symmetrical and longer than the halogen bond distances in the corresponding molecular 2:1 adducts. The C–I bond distances of 2.0827(5) Å and 2.0814(6) Å are in the similar range to **1**, **2** and **3**. The I1–O1–I2 angle of 108.104(16)° is significantly widened by comparison to **1** and **2**.

In molecular halogen bond **5**, the halogen bond distance I1···O1 is 2.846(2) Å and shows a contraction relative to the sum of the van der Waals radii of 20%,^25^ in a similar range as the molecular thf adduct **3** and again shorter than the supramolecular 1:1 bifurcated halogen bond in **4**. Again, the C–I bond distance is slightly elongated to 2.082(3) Å and in the same range as in all structures reported here.

**Figure 5:**
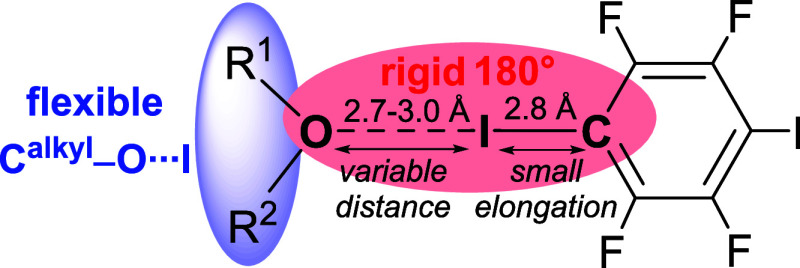
Overview on geometric parameters obtained from structures **1**–**5**.

From the geometrical features presented, first conclusions can be drawn: whereas the properties around the iodine center are rather well-determined with a rigid O···I–C angle of around 180° and a small elongation of the C–I bond distance to about 2.08 Å, the geometric features around the oxygen center are far more flexible (Figure 5). Molecular unbranched as well as supramolecular oxygen-bifurcated acceptor patterns are observed, and O···I distances (2.78 to 2.96 Å) and I···O···I angles in the supramolecular bifurcated halogen bonds cover a wider range. This flexibility is significantly more pronounced than for N-accepting halogen bonds.^9,10^

Further studies in solution phase by NMR titration were conducted (for more information, see SI). However, at room temperature, the halogen bond interaction cannot be detected. By lowering the temperature, the formation of solids is observed, so that the NMR experiments cannot be carried out anymore.

To put the obtained structures in context of the literature-known halogen bonds with ethers, a CSD database search was conducted (for more information, see SI).^24^ Out of all oxygen halogen bonds to 1,4-diiodotetrafluorobenzene or iodopentafluorobenzene, pyridine oxide or carboxylate functional groups yield structures with the shortest O···I distances and presumably the strongest halogen bonds. 112 halogen bond structures with a monosubstituted iodine to oxygen ether were found. Halogen bond adducts of ethers and 1,4-diiodotetrafluorobenzene or iodopentafluorobenzene can mostly be characterized in two categories: aryl-ether and cyclic ether like morpholine, 1,4-dioxane or thf. Dialkyl ether as well as bifurcating ethers have not yet been reported, and only a small number of simple structures with no further interactions than halogen bonds are available.

**Figure 6:**
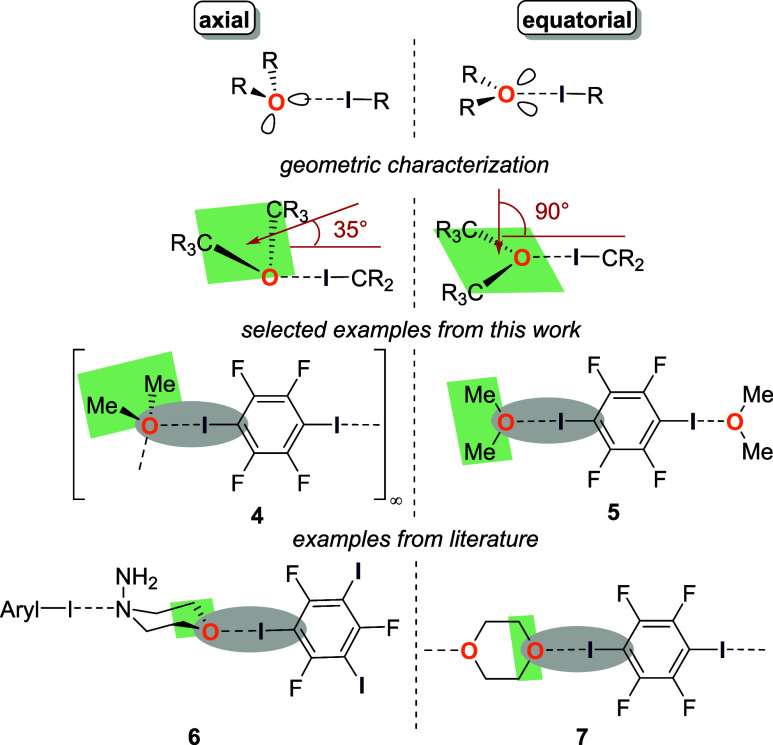
Literature-known orientations of ether molecules in halogen bonds, shown on two examples **6** and **7**, compared to selected examples from this work and illustration of angle between normal of “ether”-plane (C–O–C) and C–I bond (R = H, organic substituent).^5,15^

In general, two different arrangements of ether molecules were presented, namely in axial or equatorial positions (Figure 6).^5,14^ These can be characterized by the angle between the normal of the “ether”-plane (O1 and its two adjacent carbon atoms) and the C–I bond. This angle should be 35° for the axial arrangement in an idealized tetrahedral geometry, while for the equatorial arrangement the angle should be 90°. For the presented oxygen-bifurcated structures **1**, **2** and **4** the angles are 50.7, 36.9, and 33.29° respectively, so that this structure motif is characterized by an axial arrangement, while the bicentered halogen bond adducts **3** and **5** with an angle of 76.1 and 84.5° can easily be identified as an equatorial arrangement (see Figures 2, 3, and 4).

The structural flexibility of etheric oxygen centers in halogen bonds has already been indicated in the literature-known structures, however, the possibility of the same ether to form an adduct in both structural motifs to the same halogen bond donor has not been reported before to the best of our knowledge. By isolation of the highly sensitive and temperature-labile compounds **2**–**5**, this was realized.

In the preceding sections, we have used single crystal X-ray diffraction to investigate the geometric aspects of halogen bonds to ether molecules. Under favorable conditions, X-ray diffraction may go beyond this routine technique and provide an experimental picture of the electron density at subatomic resolution. If flawless crystals are available, redundant low-temperature data at high resolution allows to extract information about the nature of intramolecular bonds and intermolecular interactions.

Our main goal was to gain a deeper insight into the underlying bonding principles of the etheric O···I halogen bond. What is the interplay between the rigid and almost linear O···I–C arrangement and the pronounced flexibility of distances and C^alkyl^-O···I angles required to accommodate the different structural motifs in the solid state of **1**, **2**, **3**, **4** and **5**?

In particular, results from preceding electron density studies of halogen bonds with *N*-Lewis bases^9,10^ or electron density studies and geometrical flexibility of etheric ligands in coordinating modes to lithium alkyls^28^ raised the question of parallels and the behavior of etheric oxygen in halogen bonds. For the unprecedented supramolecular oxygen-bifurcated ether···halogen bonds, an understanding of the electronic situation is outright mandatory.

**Figure 7:**
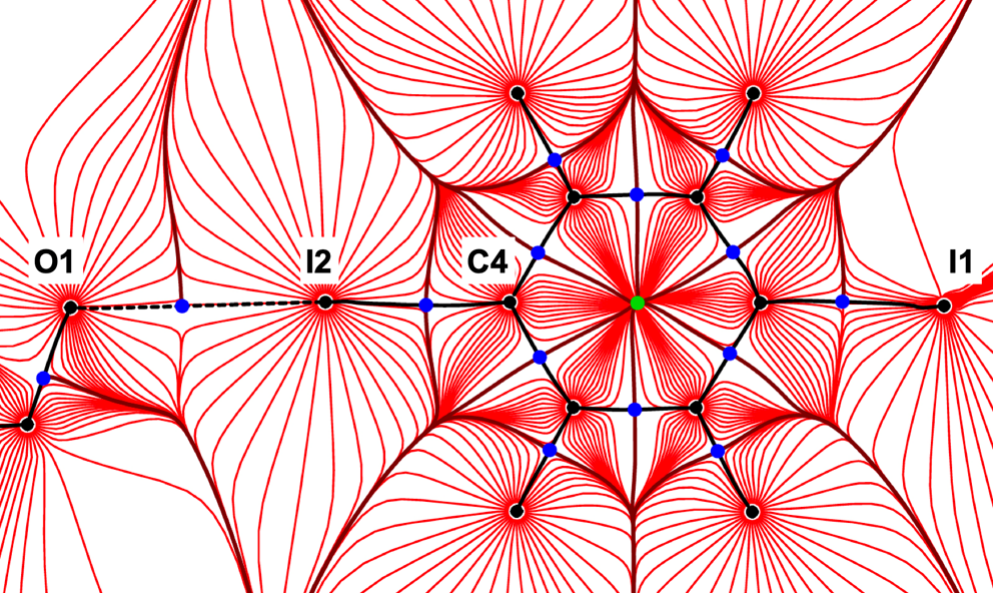
Trajectory plot of the gradient of the electron density; bond paths are shown as black lines, nuclear attractors as black, bond critical points as dark-blue and ring critical points as green solid circles. Key quality criteria for the refinement: resolution 1.2 Å^–1^, 16855 observations, 530 variables, wR2 at convergence 0.0292; max./min./mean residual electron density 0.547/–0.538/0.082 eÅ^–3^.

Excellent crystals were available for **4**, and the diffraction data were refined^29^ based on an atom-centered multipole model.^30^ Details concerning data completeness and quality have been compiled in the Supporting Information. The resulting electron density was analyzed following Baders Quantum Theory of Atoms in Molecules (QTAIM).^31^Figure 7 depicts the gradient of the electron density in the plane of the halogen donor molecule, together with its critical points; key quality criteria of the refinement are provided in the caption, and a comprehensive table of refinement results is provided in the Supporting Information.

We are aware of only a few experimental electron density studies involving short O···I contacts, but the results obtained for **4** fit well into this admittedly limited context. The almost linear bond path associated with the short O1···I2 distance in Figure 7 is characterized by an electron density in the bond critical point (ρ_bcp_) of 0.160(4) eÅ^–3^. Bianchi et al. had reported 0.2 eÅ^–3^ for a shorter contact involving a dipyridyl dioxide,^32^ and values around 0.1 eÅ^–3^ were encountered for longer contacts between 3 and 3.2 Å.^33^ A table summarizing the electron density and its derived properties in all bond critical points in **4** is available in the Supporting Information.

**Figure 8:**
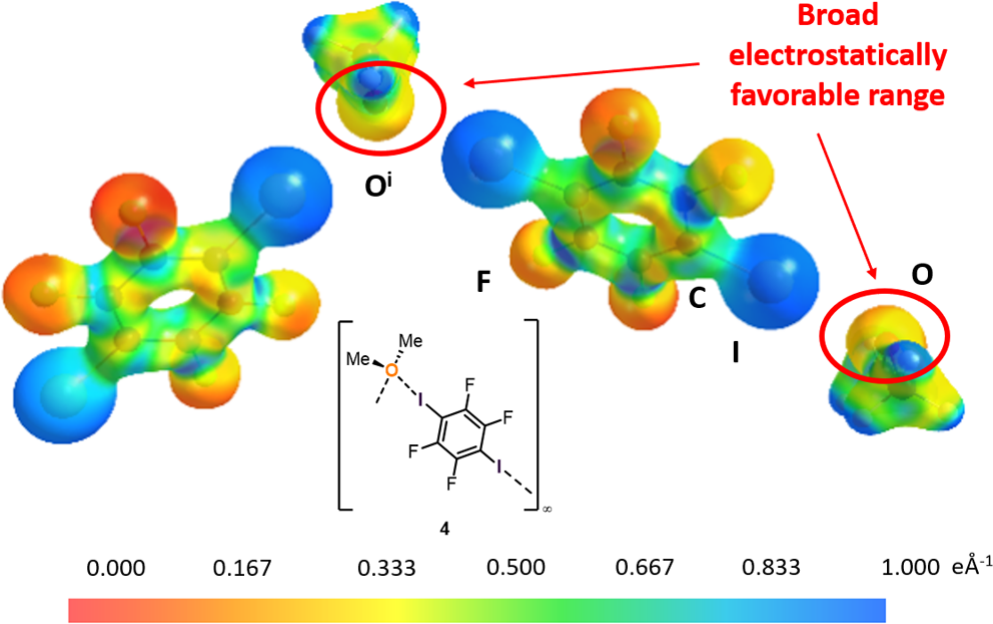
Experimentally derived electrostatic potential [eÅ^–1^] for **4** mapped on an electron density isosurface of 0.5 eÅ^–3^. Two symmetry equivalent halogen bond adducts from the extended chain along [0 1 0] are shown.^30^

The very concept of the σ-hole emphasizes the relevance of electrostatic contributions to the halogen bond. Mappings of the electrostatic potential (ESP) on isodensity surfaces provide an intuitive understanding of electrostatic complementarity between the interacting partners^34^ in a directional contact such as a halogen bond. Figure 8 depicts the experimentally derived electrostatic potential for a section of the extended chains in **4**. The oxygen center displays a wide range for favorable interactions with more positively charged regions of neighbor molecules, e.g., on adjacent iodine atoms. One or two iodine centers may address this range in a flexible manner rather than interact with one or two electron lone pair of the oxygen; thus the flexible geometry about the halogen bond acceptor finds an intuitive explanation.

Energy densities in the bond critical point can be used to further classify secondary interactions. The kinetic energy density *G* was derived with the procedure suggested by Abramov,^35^ and the potential energy density *V* was obtained according to the local virial theorem.^36^ The total energy density *E* as the sum of the (positive) kinetic energy density *G* and the (negative) potential energy density *V* is associated with negative values in the bcps of covalent bonds.^37^ For secondary interactions, *E* usually adopts positive values, with a few exceptions for very strong hydrogen bonds^38^ or very short halogen bonds with significant covalent contributions.^39^Table S13 in the Supporting Information shows that the O···I halogen bonds in **4** are characterized by a positive Laplacian and by a slightly positive total energy density in their bond critical points. These derived properties of the experimental electron density support our classification of the contacts between ether oxygen and a strong halogen bond donor as essentially electrostatic interactions.

A secondary geometric effect supports this assignment: The adjacent C–I bond responsible for the σ-hole is significantly elongated with respect to the “free” halogen bond donor, but this effect is clearly less pronounced than in the stronger and shorter N···I halogen bonds with their more directed lone pair at the nitrogen center.^9,10^

Our analysis of the bonding situation relies on experiment, and it is tempting to repeat this analysis with theoretical methods and compare the results. Oliveira et al. have suggested coupled-cluster theory for the description of halogen bonds in discrete aggregates^40^ and Arhangelskis et al. have tested different types for dispersion correction in periodic DFT calculations for extended structures.^41^ We frankly admit that a concomitant attempt to reproduce the geometry of moderately strong intermolecular interactions in discrete molecular AND in extended structures is beyond our expertise. It might be an attractive aim for a group of theoretical chemists to identify a method which can reliably reproduce both structural motifs shown here.

## Conclusions

To conclude, we presented studies on interactions between iodine in a strong halogen bond donor and oxygen in popular (mtbe, thf) and even prototypic (dme) ethers as acceptors. Interestingly, two different structural motifs were encountered even when the same ether molecule was involved. One motif displayed a discrete molecular adduct in a 2:1 ratio of ether to iodine, with a bicentered halogen bond. The second and unexpected motif is characterized by bifurcating ether oxygen centers forming a supramolecular chain-extended adduct. To the best of our knowledge, such a bifurcating behavior has not yet been reported for ether oxygen centers in halogen bonds. Both motifs can be obtained selectively by adjusting the stoichiometric ratio of the halogen bonding partners. For ethers with variable steric requirements, diffraction experiments at standard resolution highlight a broad range of O···I distances and C^alkyl^–O···I angles and thus pronounced flexibility of the oxygen center, whereas the σ-hole-related O···I–C angle faithfully adopts values close to 180°.

In the solids presented here, O···I interactions represent the only short directional contacts; this fact allows a deeper insight into the halogen bond. For the supramolecular bifurcating dme-system **4** an experimental electron density study was conducted based on high resolution single crystal X-ray diffraction data. The properties derived from the experimental electron density such as energy densities in the bond critical points and the Laplacian suggest an essentially electrostatic character for the halogen bond. The electrostatic potential shows a wide negatively charged region at the ether oxygen atom suitable for interaction with the (positive) σ-hole(s) of one or two XB donors. The oxygen center can therefore engage in unbranched or oxygen-bifurcating halogen bonds and shows high geometric flexibility in the latter, with I···O···I angles between 89° in **2** and 108° in **4**. The acceptor-bifurcated motif can thus be perceived as the electrostatically favorable interaction between an electronegative oxygen and the σ-holes of two I XB donor atoms, in other words as a tricentered halogen bond. Different from *N*-containing systems, this broad flexibility of the geometry around oxygen in principle allows for a large variety of functionalization and applications in various contexts.

Our studies highlight the relevance of weak and moderately strong intramolecular halogen bonds involving simple and commonly used or prototypic ethers. Through interaction with 1,4-diiodotetrafluorobenzene crystals based on halogen bonds can be isolated. They owe their existence to favorable overall energy in the solid state at low temperatures. Analogous interactions are feasible in more complex solids with additional interactions such as biological systems.

## Supporting Information Available

Crystallographic data (CIF)
Information on crystallization procedures, NMR titration experiments, X-ray crystallographic analyses, and experimental electron density (PDF)

## Supplementary Material

ao4c05124_si_001.cif
