# Supplementary material for: The Halogen Bond to Ethers - Prototypic Molecules and Experimental Electron Density
Source: ACS Omega. 2024 Aug 5;9(32):35037–45. doi: 10.1021/acsomega.4c05124 (PMC11325402; doi:10.1021/acsomega.4c05124)
Supplement: Supplementary file 2 — ao4c05124_si_002.pdf [file ao4c05124_si_002.pdf]

# Supporting Information

# The Halogen Bond to Ethers – Prototypic Molecules and Experimental Electron Density

Annika Schmidt,[a] Anna Krupp,[a] Johannes Kleinheider,[a] Tamara M. L. Binnenbrinkmann,[a] Ruimin Wang,[b,c] Ulli Englert,*[b,c] Carsten Strohmann*[a]

[a] A. Schmidt, Dr. A. Krupp, Dr. J. Kleinheider, T. M. L. Binnenbrinkmann, Prof. Dr. C. Strohmann, Inorganic Chemistry, TU Dortmund University, Otto-Hahn-Straße 6, 44227 Dortmund, Germany.
E-mail: carsten.strohmann@tu-dortmund.de

[b] Dr. R. Wang, Prof. Dr. U. Englert, RWTH Aachen University , Institute of Inorganic Chemistry, Aachen, Germany
E-mail: ullrich.englert@ac.rwth-aachen.de

[c] Dr. R. Wang, Prof. Dr. U. Englert, Shanxi University, Key Laboratory of Materials for Energy Conversion and Storage, Institute of Molecular Science, Taiyuan, Shanxi 030006, People's Republic of China

# Table of Contents

**Experimental Procedures** 2

1 General Remarks 2

2 Crystallization of Compounds 1-5 3

3 NMR Titration Experiments 5

**Results and Discussion** 8

1 X-Ray Crystallographic Analyses 1-5 8

2 CSD Database Search 13

3 Experimental Electron Density Study 15

# Experimental Procedures

## 1 General remarks

All chemical syntheses with air-sensitive reagents were carried out in dried, oxygen-free solvents under an inert gas atmosphere of argon (Argon 5.0 from *Messer Griesheim GmbH*). The standard glass apparatus used was baked out in an evacuated state (10–2 bar) before working with oxygen- or moisture-sensitive compounds. The solvents used were dried, purified, distilled and stored under argon atmosphere according to standard procedures. All reagents used, unless otherwise stated, were hand-delivered products without further purification. The deuterated solvents used for the NMR spectroscopy were products of *Eurisotop* and Deutero. All other reagents were products of the companies *Sigma Aldrich GmbH, FLUKA, Alfa Aesar, ABCR* and *Acros Organics*. The NMR solvent benzene-d6 was also stored over sodium wires. The NMR spectra were measured on a 500 MHz Bruker Avance NEO, 600 MHz Bruker Avance III HD and 500 MHz Agilent Technologies DD2 spectrometer at 25 °C. Chemical shifts (δ in ppm) are referred to tetramethylsilane (TMS), with the deuterium signal of the solvent serving as internal lock. The chemical shift data in ppm refer to the δ-scale. Spin-spin coupling constants (*J*) were given in hertz (Hz) ($^{n}J_{XY}$: coupling of observed nucleus X with a n bond distant nucleus Y). The following abbreviations were used to reflect multiplicities and signal shape: s = singlet, d = doublet, q = quartet, m = multiplet, br = broad signal. $^{1}$H-NMR spectra: Locking substances (internal standard): Benzene (δ = 7.16), chloroform (δ = 7.27). The number of hydrogen atoms per signal was determined by comparing the relative signal intensities. Data collections for the compounds **1**-**5** were conducted on a Bruker D8 Venture four-circle diffractometer by *Bruker AXS GmbH* using a PHOTON II CPAD detector by *Bruker AXS GmbH*. X-ray radiation was generated by a microfocus source IμS Mo by *Incoatec GmbH* with HELIOS mirror optics and a single-hole collimator by *Bruker AXS GmbH*. For the data collection, the programs APEX 4 Suite with the integrated programs SAINT (integration) and SADABS (absorption correction) by *Bruker AXS GmbH* were used. Using Olex2.1, the structures were solved with the ShelXT 2 structure solution program by Intrinsic Phasing and refined with the XL 3 refinement package using Least Squares minimization. For the selection of air and moisture sensitive crystals the X-TEMP 2 4 system was used in combination with a SMZ1270 stereomicroscope from *Nikon Metrology GmbH*. MicroGrippers from MiTeGen were used for mounting.

Atomic coordinates and other structural parameters of **1**-**5**, have been deposited with the Cambridge Crystallographic Data Centre (CCDC numbers 2334578 (for **1**), 2334577 (for **2**), 2334576 (for **3**), 2334579 (for the independent atom model for **4**), 2334580 (for the multipole model for **4**), and 2334581 (for **5**).

## 2 Crystallization of Compounds 1-5

### 2.1 Crystallization of 1,4-diiodotetrafluorobenzene halogen bond adduct with mtbe (1)

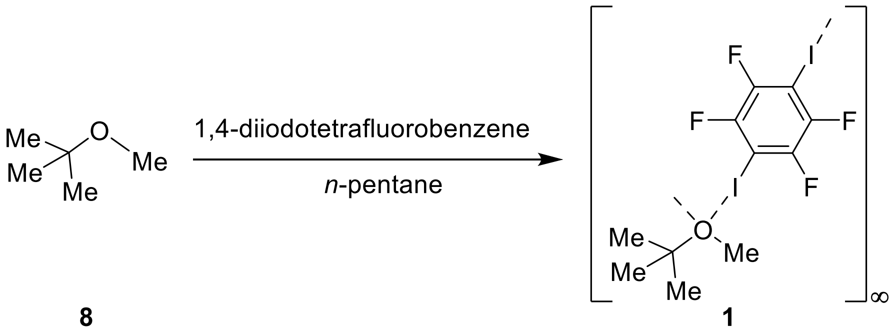


**8**
$C_5H_{12}O$
88.15 g mol$^{-1}$

**1**
$C_{11}H_{12}F_4I_2O$
490.02 g mol$^{-1}$

In the absence of water and oxygen, methyl *tert*-butyl ether (**8**, 70.5 mg, 95.3 µL, 0.80 mmol, 2.0 eq.) and 1,4-diiodotetrafluorobenzene (161 mg, 0.40 mmol, 1.0 eq.) were dissolved in 0.50 mL *n*-pentane. Compound **1** crystallized at –80 °C as colorless blocks.

### 2.2 Crystallization of polymeric 1,4-diiodotetrafluorobenzene halogen bond adduct with thf (2)

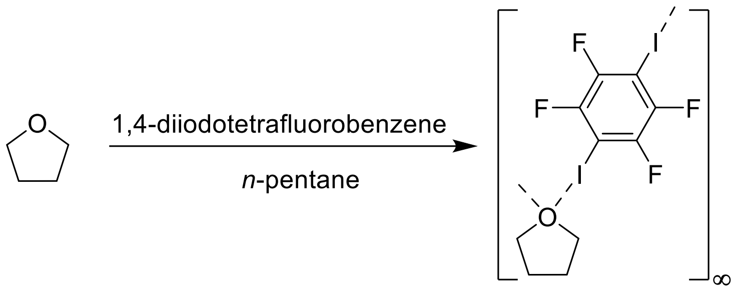


**9**
$C_4H_8O$
72.11 g mol$^{-1}$

**2**
$C_{10}H_8F_4I_2O$
473.98 g mol$^{-1}$

In the absence of water and oxygen, tetrahydrofuran (**9**, 28.8 mg, 32 µL, 0.40 mmol, 1.0 eq.) and 1,4-diiodotetrafluorobenzene (163 mg, 0.41 mmol, 1.0 eq.) were dissolved in 0.50 mL *n*-pentane. Compound **2** crystallized at –80 °C as colorless needles.

### 2.3 Crystallization of molecular 1,4-diiodotetrafluorobenzene halogen bond adduct with thf (3)

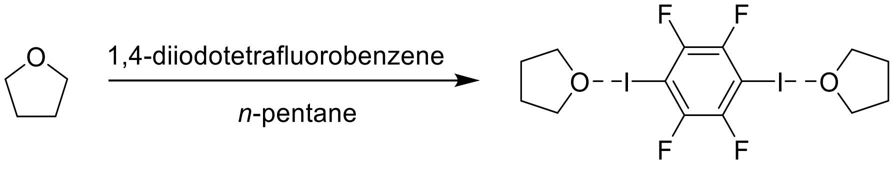


**9**
$C_4H_8O$
72.11 g mol$^{-1}$

**3**
$C_{14}H_{16}F_4I_2O_2$
546.08 g mol$^{-1}$

In the absence of water and oxygen, tetrahydrofuran (**9**, 89 mg, 0.1 mL, 1.23 mmol, 3.0 eq.) and 1,4-diiodotetrafluorobenzene (163 mg, 0.41 mmol, 1.0 eq.) were dissolved in 0.50 mL *n*-pentane. Compound **3** crystallized at –80 °C as colorless blocks.

### 2.4 Crystallization of polymeric 1,4-diiodotetrafluorobenzene halogen bond adduct with dme (4)

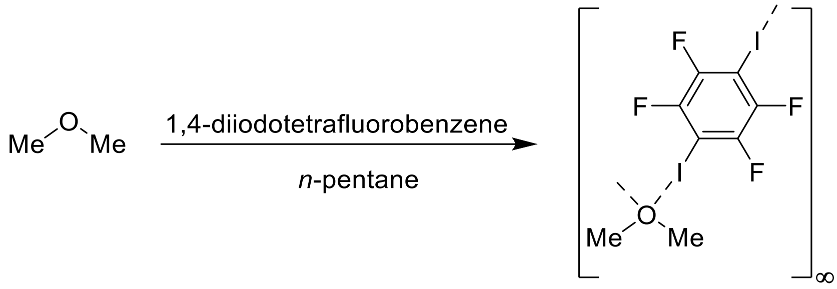


**10** $C_2H_6O$ 46.07 g mol$^{-1}$ **4** $C_{10}H_8F_4I_2O$ 447.94 g mol$^{-1}$

In the absence of water and oxygen, 0.1 mL dimethyl ether (**10**, 74.0 mg, 1.61 mmol, 3.3 eq.) and 1,4-diiodotetrafluorobenzene (195 mg, 0.49 mmol, 1.0 eq.) were dissolved in 0.50 mL *n*-pentane. Compound **4** crystallized at –80 °C as colorless needles.

### 2.5 Crystallization of molecular 1,4-diiodotetrafluorobenzene halogen bond adduct with dme (5)

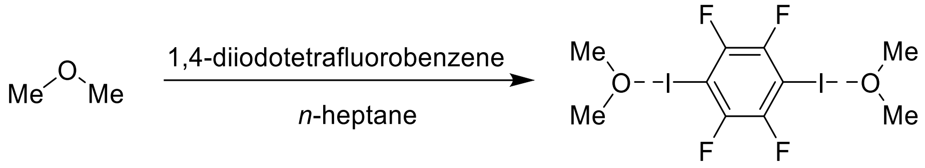


**10** $C_2H_6O$ 46.07 g mol$^{-1}$ **5** $C_{10}H_{12}F_4I_2O_2$ 494.01 g mol$^{-1}$

In the absence of water and oxygen, dimethyl ether (**10**, 148 mg, 0.20 mL, 3.21 mmol, 6.6 eq.) and 1,4-diiodotetrafluorobenzene (201 mg, 0.49 mmol, 1.0 eq.) were dissolved in 0.50 mL *n*-heptane. Compound **5** crystallized at –80 °C as colorless blocks.

Up to 3.3 eq. of dimethylether, compound **4** was obtained. When using more equivalents compound **5** was formed.

Even though dimethyl ether is gaseous at room temperature, it can be well handled below its boiling point of –24 °C. Therefore, we condensed the gas in a Schlenk finger at –80 °C that is also storable at –80 °C. When the Schlenk finger is at –80°C itself, the system has cooled sufficiently to be easily dosed by syringe.

As the obtained crystals are characterized by high sensitivity regarding temperature, no Raman spectra were able to be conducted.

The differentiation of the molecular from the polymeric compound in the case of thf and dme has been done visually, as one compound crystallizes as needles and the other one as blocks. Furthermore, the molecular compounds **3** and **5** are characterized by an even higher instability, making the differentiation possible.

## 3 NMR Titration Experiments

To check the presence of a halogen bond in solution, NMR titration experiments of the thf system with pentafluoroiodobenzene were conducted. Pentafluoroiodobenzene was chosen as a test system due to the clearer evaluation of the obtained NMR spectra in contrast to 1,4-diiodotetrafluorobenzene. Thereby, one halogen bond partner was defined as host and a "host solution" with a constant concentration of 20 mM in 2 mL of cyclohexane-$d_{12}$ was prepared. Subsequently, 400 µmol of the other halogen bond partner (defined as guest) was added to 1 mL of this "host solution". This solution is defined as "guest solution" and used for the titration. Out of the remaining host solution 0.6 mL were transferred into an NMR tube and a defined amount of NMR standard (2µL to 10 µL tetramethylsilane for $^{1}$H-NMR titrations and 2 µL to 10 µL hexafluorobenzene for $^{19}$F-NMR titrations) was added.

For the titration itself, the prepared sample was analyzed by NMR; after the measurement a defined amount of "guest solution" was added and analyzed by NMR again etc.

This procedure was repeated with two identical samples in parallel. Also, both halogen bond donor and acceptor were identified as host or guest in two distinct experiments.

a) thf as host, pentafluoroiodobenzene as guest

The obtained change of the $^{1}$H-NMR shift $\delta$ of the $CH_2$-thf protons in dependency on the concentration of the halogen bond donor pentafluoroiodobenzene (guest) is plotted in Figure S1. The experiment was carried out twice, which is reflected by the blue and red markers in Figure S1.

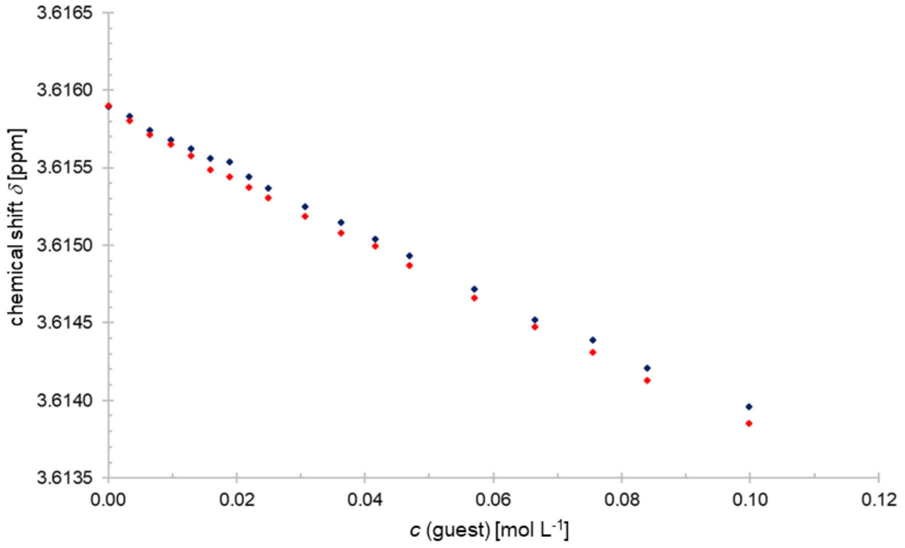


**Figure S1.** Titration curve of a thf (host)/pentafluoriodobenzene (guest) mixture in cyclohexane-$d_{12}$ at room temperature. The chemical shift $\delta$ is plotted against the guest concentration $c$. The thf concentration was maintained constant. Blue and red markers indicate two different experiments.

The chemical shift of the $CH_2$-thf protons only changes slightly and linearly. The expected logarithmic behavior for a positive interaction cannot be observed.[1] The slight change of shift may be explained by a change of polarity of the solution due to titration of pentafluororbenzene.

b) pentafluoroiodobenzene as host, thf as guest

The obtained change of the $^{19}$F-NMR shift $\delta$ of the *ortho*-fluorine atoms of pentafluoroiodobenzene in dependency on the concentration of the halogen bond acceptor thf (guest) is plotted in Figure S2. The experiment was carried out twice, which is reflected by the blue and red markers in Figure S2.

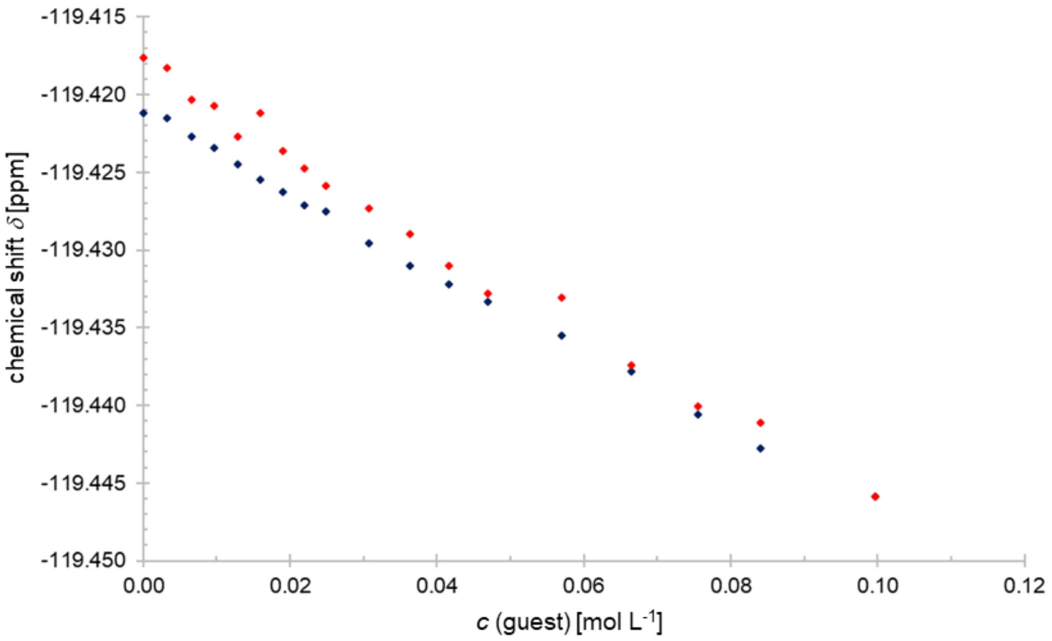


**Figure S2.** Titration curve of a pentafluoriodobenzene (host)/thf (guest) mixture in cyclohexane-$d_{12}$ at room temperature. The chemical shift $\delta$ is plotted against the guest concentration *c*. The pentafluoroiodobenzene concentration was maintained constant. Blue and red markers indicate two different experiments.

Again, only a slight and linear change is observed. No halogen bond interaction was detected.

The analysis of the halogen bond adduct **3** also showed no proof of halogen bond formation. From comparable systems with *N*-halogen bond acceptors, it is known that the $^{19}$F-NMR signal of 1,4-diiodotetrafluorobenzene splits into a multiplet due to a desymmetrization because of the halogen bond formation. However, this can not be observed (Figure S3).

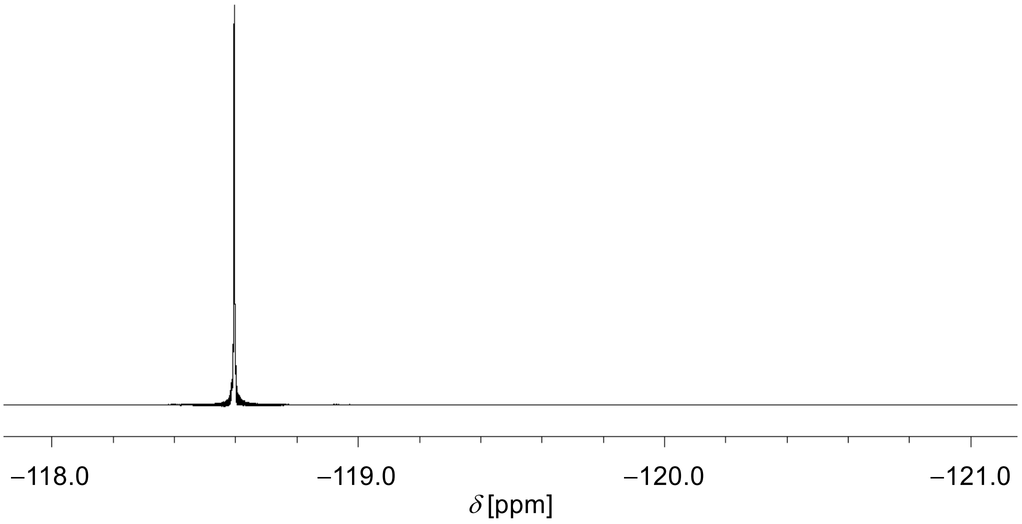


**Figure S3.** Excerpt of $^{19}$F-NMR spectra of **3** in solution.

Even though the halogen bond was not able to be detected in solution at room temperature, its relevance also in liquid phase should not be underestimated. By using ethers as solvents, their concentration is much higher than in the conducted NMR titration experiments, also, by lowering the temperature, the halogen bond interactions are expected to become more dominant.

NMR titrations in cyclohexane below room temperature were not conducted due to the high melting point of cyclohexane-$d_{12}$. The fast crystallization of the halogen bond adduct in *n*-pentane leads to solid formation during cooling.

## Results and Discussion

### 1 X-Ray Crystallographic Analyses

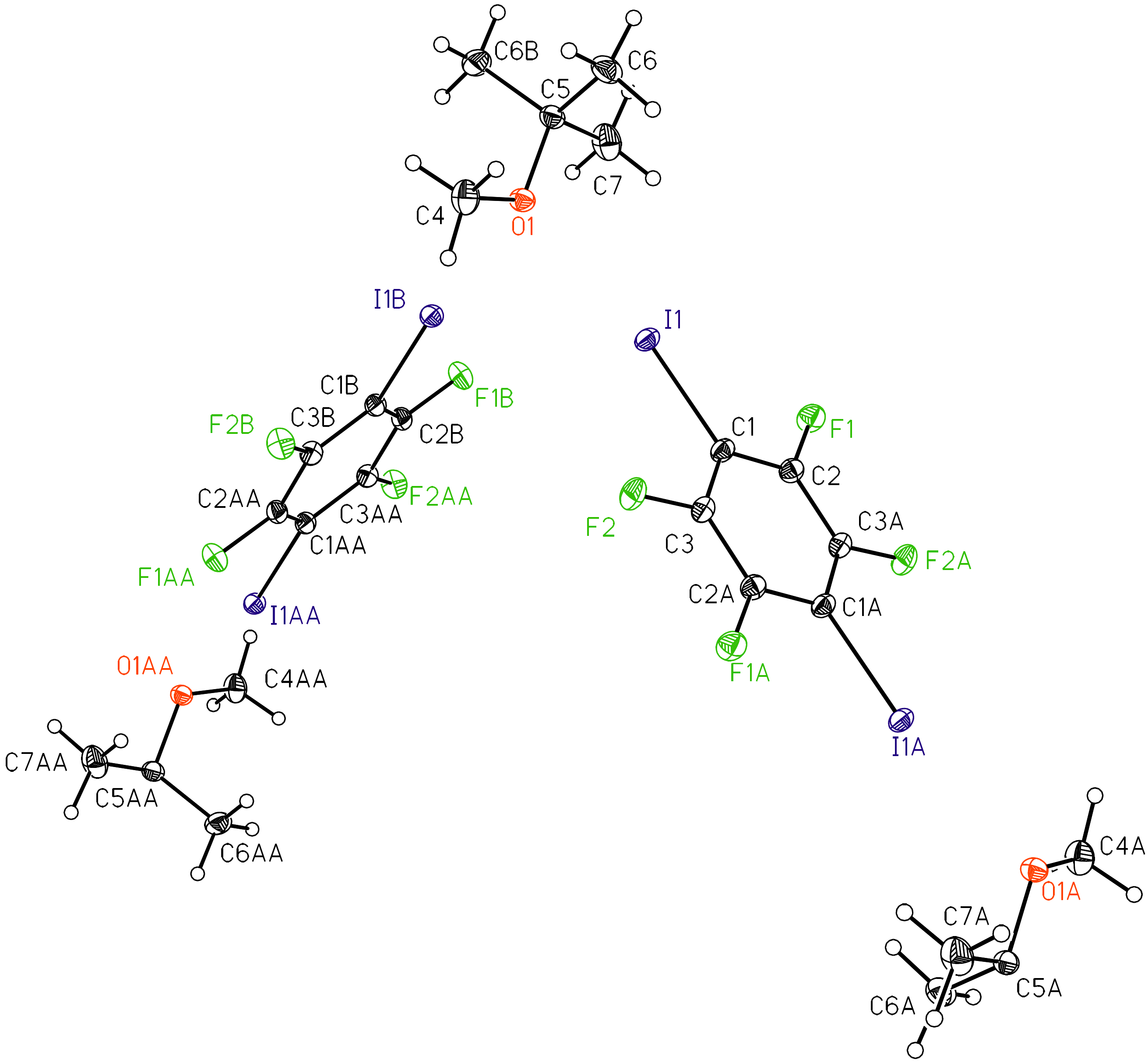


**Figure S4.** Displacement ellipsoid plot of the structure of the halogen bond adduct **1** in the crystal with ellipsoids drawn at 50% probability. Numbering of hydrogen atoms omitted for clarity. Selected distances [Å] and [°]: I1–O1 2.9699(9), C1–I1 2.0847(11), C1–I1–O1 172.44(4), I1–O1–I1B 91.60(4). CCDC deposition number 2334578.

**Table S1.** Crystallographic data and structural refinements for **1**.

| | **1** |
|---|---|
| Empirical formula | $C_{11}H_{12}F_4I_2O$ |
| Formula weight [g·mol$^{-1}$] | 490.01 |
| Temperature [K] | 100.00 |
| Crystal system | orthorhombic |
| Space group | *P*nma |
| a [Å] | 13.0219(6) |
| b [Å] | 17.2708(9) |
| c [Å] | 6.2616(3) |
| $\alpha$ [°] | 90 |
| $\beta$ [°] | 90 |
| $\gamma$ [°] | 90 |
| Volume [Å$^3$] | 1408.23(12) |
| Z | 4 |
| $\rho_{calc}$ [g/cm$^3$] | 2.311 |
| μ [mm$^{-1}$] | 4.497 |
| F(000) | 912.0 |
| Crystal size [mm$^3$] | 0.202 × 0.161 × 0.109 |
| Radiation | MoK$\alpha$ ($\lambda$ = 0.71073) |
| 2Θ range for data collection [°] | 4.718 to 80.586 |
| Index ranges | $-23 \le h \le 23$<br>$-31 \le k \le 31$<br>$-11 \le l \le 11$ |
| Reflections collected | 106090 |
| Independent reflections | 4548<br>[$R_{int}$ = 0.0463, $R_{sigma}$ = 0.0140] |
| Data/restraints/parameters | 4548/0/89 |
| Goodness-of-fit on F$^2$ | 1.041 |
| Final R indexes [I>=2σ (I)] | $R_1$ = 0.0200, w$R_2$ = 0.0426 |
| Final R indexes (all data) | $R_1$ = 0.0261, w$R_2$ = 0.0455 |
| Largest diff. peak/hole [e Å$^{-3}$] | 1.24/-0.86 |

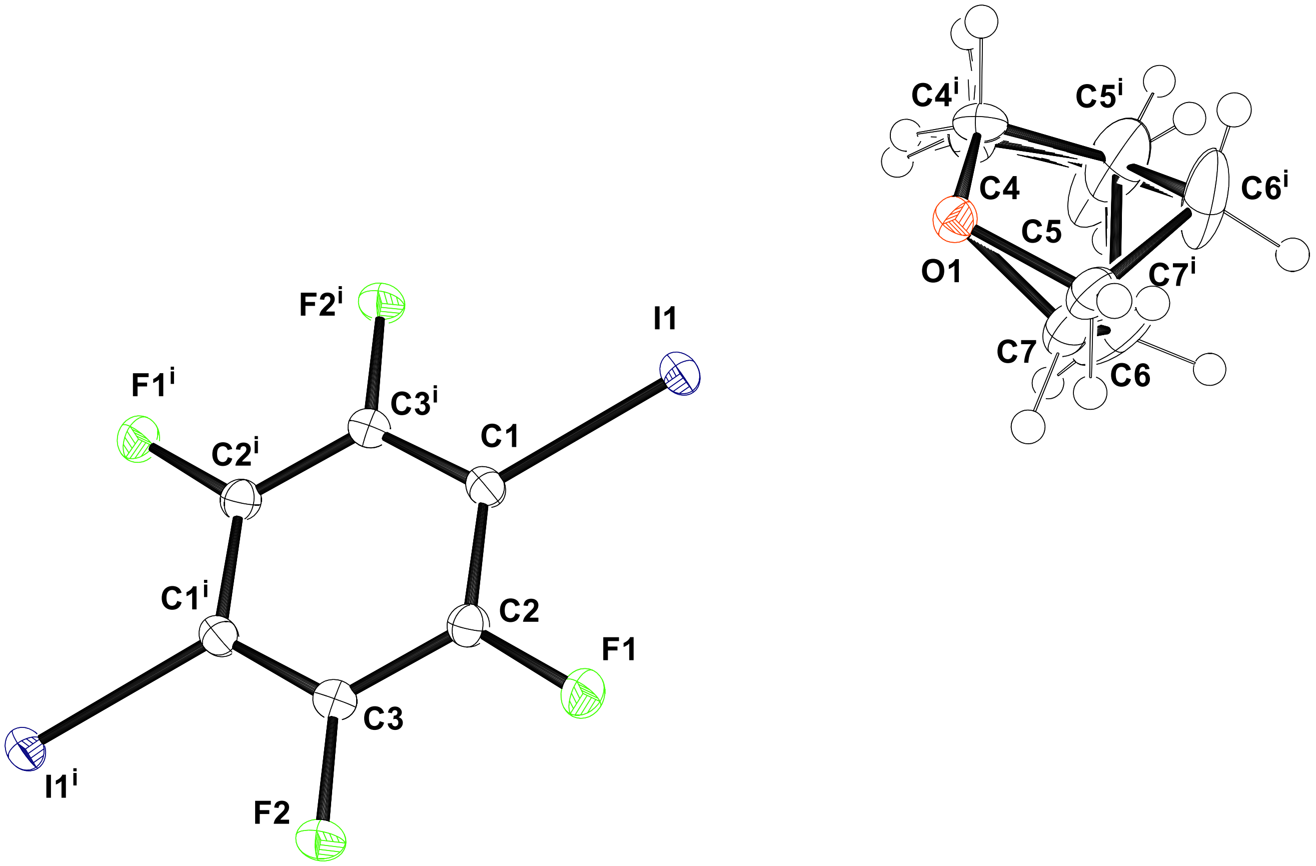


**Figure S5.** Displacement ellipsoid plot of the structure of the halogen bond adduct **2** in the crystal with ellipsoids drawn at 50% probability. Numbering of hydrogen atoms omitted for clarity. Selected bond lengths [Å] and angles [°] for **2**: I1···O1 2.9035(13), I1–C1 2.0842(16), C1–I1···O1 176.41(6), $I1^{i}$···O1···I1 89.44(5); angle of normal of area A(O1,C4,C7) and bond C1–I1 36.9(13); i = 1–x,1–y,1–z; two domains were integrated separately; TWINABS-2012/1 (Bruker,2012) was used for absorption correction; for component 1: $wR_2$(int) was 0.1209 before and 0.0553 after correction. For component 2: $wR_2$(int) was 0.1857 before and 0.0677 after correction. The Ratio of minimum to maximum transmission is 0.56. Final HKLF 4 output contains 232813 reflections, Rint = 0.0517 (135861 with I > 3sig(I), Rint = 0.0453). CCDC deposition number 2334577.

**Table S2.** Crystallographic data and structural refinements for **2**.

| | **2** |
|---|---|
| Empirical formula | $C_{10}H_8F_4I_2O$ |
| Formula weight [g·mol$^{-1}$] | 473.96 |
| Temperature [K] | 100.00 |
| Crystal system | orthorhombic |
| Space group | *P*nma |
| a [Å] | 11.9753(12) |
| b [Å] | 17.4463(17) |
| c [Å] | 6.1182(6) |
| $\alpha$ [°] | 90 |
| $\beta$ [°] | 90 |
| $\gamma$ [°] | 90 |
| Volume [Å$^3$] | 1278.2(2) |
| Z | 4 |
| $\rho_{calc}$ [g/cm$^3$] | 2.463 |
| μ [mm$^{-1}$] | 4.950 |
| F(000) | 872.0 |
| Crystal size [mm$^3$] | 0.535 × 0.154 × 0.132 |
| Radiation | MoK$\alpha$ ($\lambda$ = 0.71073) |
| 2Θ range for data collection [°] | 4.67 to 80.748 |
| Index ranges | $-21 \leq h \leq 0$<br>$-31 \leq k \leq 0$<br>$-11 \leq l \leq 0$ |
| Reflections collected | 4140 |
| Independent reflections | 4140<br>[$R_{int}$ = 0.0517, $R_{sigma}$ = 0.0101] |
| Data/restraints/parameters | 4140/0/97 |
| Goodness-of-fit on F$^2$ | 1.208 |
| Final R indexes [I>=2σ (I)] | $R_1$ = 0.0309, $wR_2$ = 0.0565 |
| Final R indexes (all data) | $R_1$ = 0.0345, $wR_2$ = 0.0578 |
| Largest diff. peak/hole [e Å$^{-3}$] | 1.79/-0.87 |

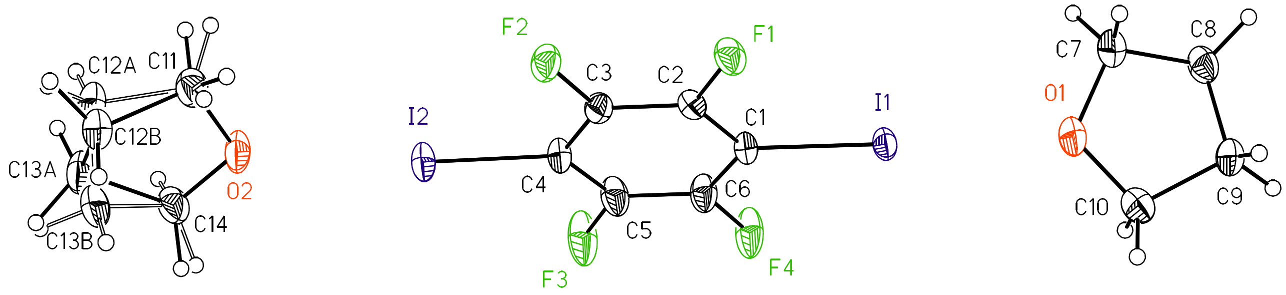


**Figure S6.** Displacement ellipsoid plot of the structure of the halogen bond adduct **3** in the crystal with ellipsoids drawn at 50% probability. Numbering of hydrogen atoms omitted for clarity. Selected distances [Å] and [°]: I1–O1 2.785(2), C1–I1 2.086(2), I2–O2 2.841(2), C4–I2 2.088(3), C1–I1–O1 177.09(9), C4–I2–O2 173.47(9). CCDC deposition number 2334576.

**Table S3.** Crystallographic data and structural refinements for **3**.

| | **3** |
|---|---|
| Empirical formula | $C_{14}H_{16}F_4I_2O_2$ |
| Formula weight [g·mol$^{-1}$] | 546.07 |
| Temperature [K] | 100.00 |
| Crystal system | triclinic |
| Space group | $P\bar{1}$ |
| a [Å] | 9.4843(4) |
| b [Å] | 9.6529(4) |
| c [Å] | 10.5569(4) |
| $\alpha$ [°] | 99.237(2) |
| $\beta$ [°] | 97.4730(10) |
| $\gamma$ [°] | 114.6070(10) |
| Volume [Å$^3$] | 846.19(6) |
| Z | 2 |
| $\rho_{calc}$ [g/cm$^3$] | 2.143 |
| μ [mm$^{-1}$] | 3.758 |
| F(000) | 516.0 |
| Crystal size [mm$^3$] | 0.527 × 0.392 × 0.234 |
| Radiation | MoK$\alpha$ ($\lambda$ = 0.71073) |
| 2Θ range for data collection [°] | 4.006 to 61.158 |
| Index ranges | $-13 \leq h \leq 13$<br>$-13 \leq k \leq 13$<br>$-15 \leq l \leq 15$ |
| Reflections collected | 52446 |
| Independent reflections | 5192<br>[$R_{int}$ = 0.0414, $R_{sigma}$ = 0.0217] |
| Data/restraints/parameters | 5192/0/218 |
| Goodness-of-fit on F$^2$ | 1.100 |
| Final R indexes [I>=2σ (I)] | $R_1$ = 0.0272, $wR_2$ = 0.0590 |
| Final R indexes (all data) | $R_1$ = 0.0348, $wR_2$ = 0.0624 |
| Largest diff. peak/hole [e Å$^{-3}$] | 1.14/-0.71 |

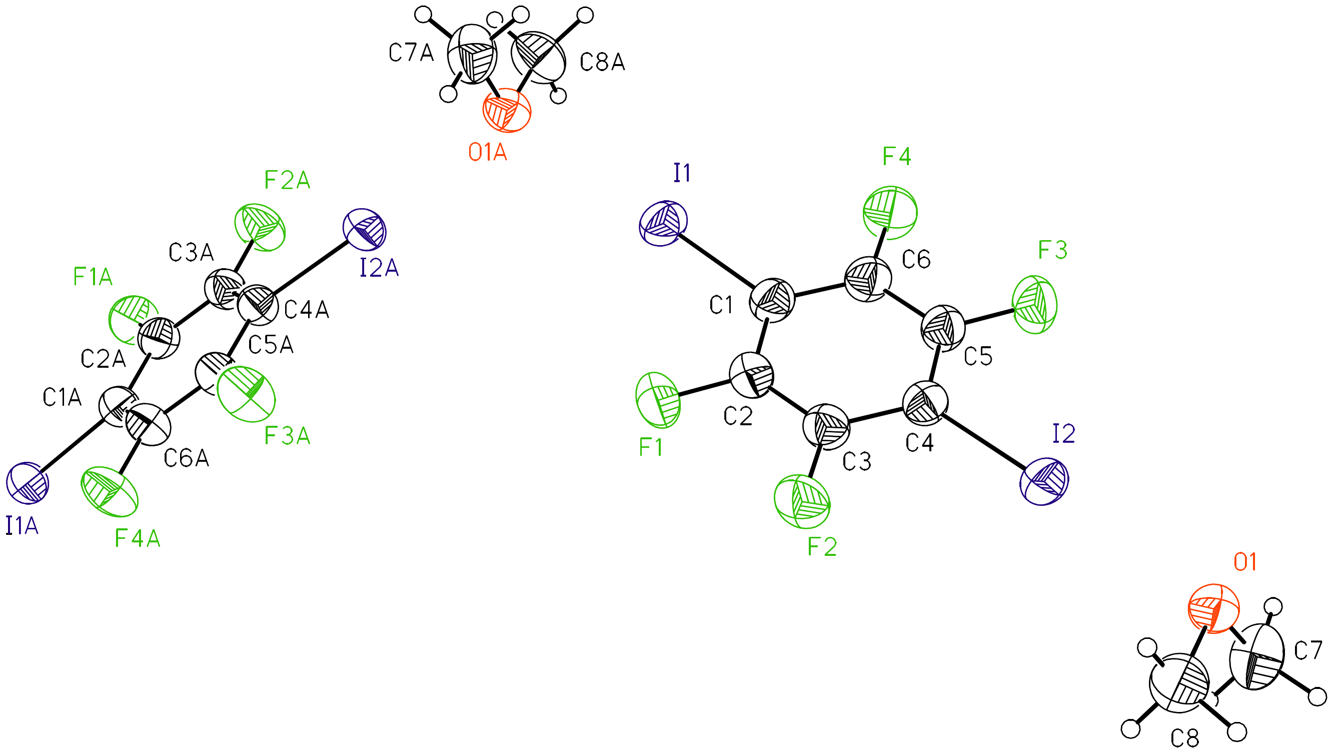


**Figure S7.** Displacement ellipsoid plot of the structure of the halogen bond adduct **4** in the crystal with ellipsoids drawn at 90% probability. Numbering of hydrogen atoms omitted for clarity. Selected distances [Å] and [°] at AIM level: I2–O1 2.8840(7), C1–I1 2.0814(8), I1–O1A 2.932(6), C4–I2 2.0836(8), C4–I2–O1 175.65(2), C1–I1–O1A 170.77(2), I1–O1A–I2A 108.10(2). CCDC deposition number 2334579 (IAM) and 2334580 (MM).

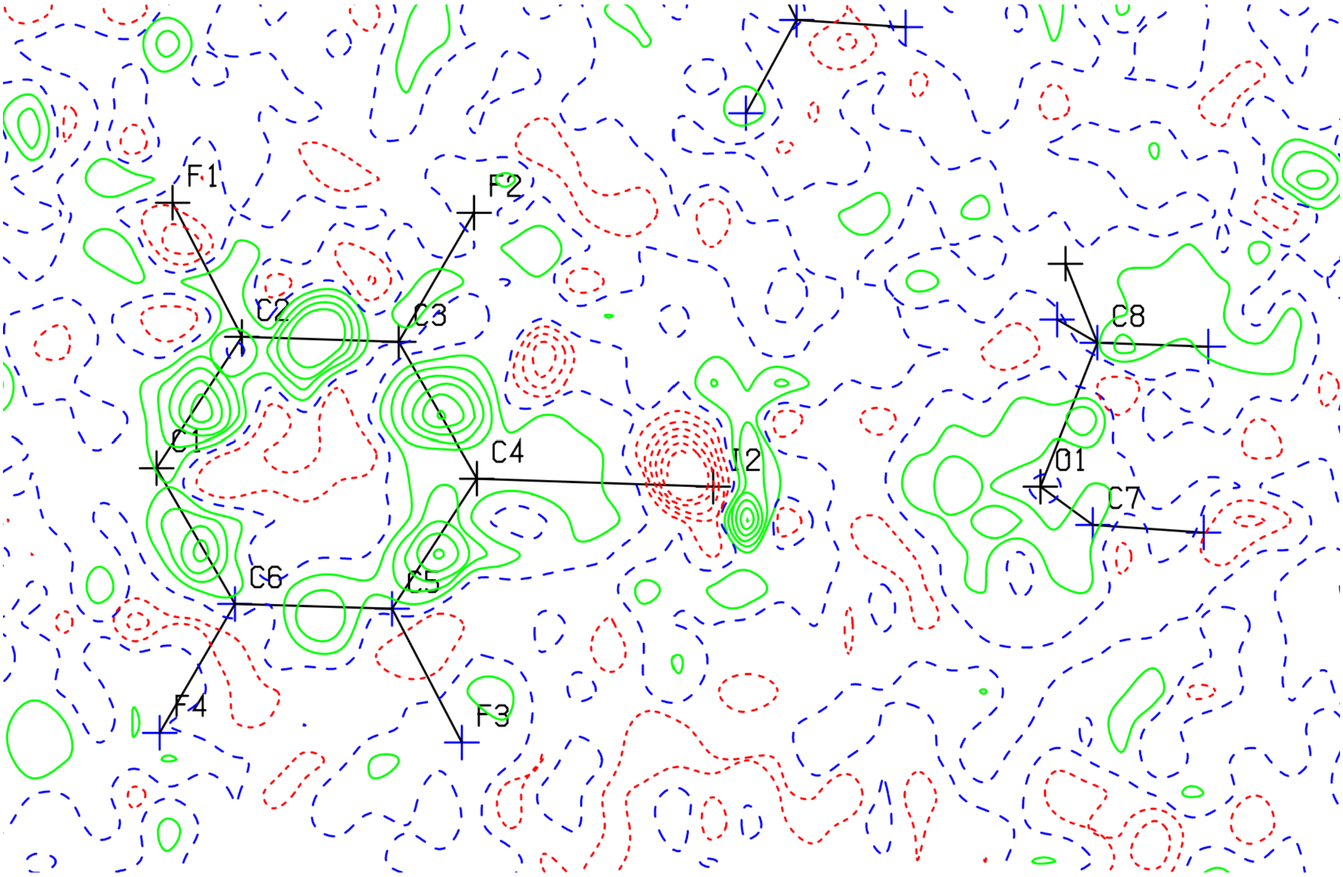


**Table S4.** Crystallographic data and structural refinements for **4**.

| | **4** |
|---|---|
| Empirical formula | $C_8H_6F_4I_2O$ |
| Formula weight [g·mol$^{-1}$] | 447.93 |
| Temperature [K] | 100(2) |
| Crystal system | monoclinic |
| Space group | $P2_1/c$ |
| a [Å] | 6.3353(10) |
| b [Å] | 21.108(3) |
| c [Å] | 9.0986(14) |
| $\alpha$ [°] | 90 |
| $\beta$ [°] | 103.9795(19) |
| $\gamma$ [°] | 90 |
| Volume [Å$^3$] | 1180.7(3) |
| Z | 4 |
| $\rho_{calc}$ [g/cm$^3$] | 2.520 |

| | |
|---|---|
| μ [mm$^{-1}$] | 5.351 |
| F(000) | 816.0 |
| Crystal size [mm$^3$] | 0.291 × 0.252 × 0.14 |
| Radiation | MoK$\alpha$ ($\lambda$ = 0.71073) |
| 2Θ range for data collection [°] | 3.86 to 116.996 |
| Index ranges | $-15 \leq h \leq 15$<br>$-50 \leq k \leq 50$<br>$-21 \leq l \leq 21$ |
| Reflections collected | 1015441 |
| Independent reflections | 17076<br>[$R_{int}$ = 0.0563, $R_{sigma}$ = 0.0077] |
| **Refinement results for the IAM** | |
| Data/restraints/parameters | 17076/0/141 |
| Goodness-of-fit on $F^2$ | 1.212 |
| Final R indexes [I>=2σ (I)] | $R_1$ = 0.0186, $wR_2$ = 0.0391 |
| Final R indexes (all data) | $R_1$ = 0.0209, $wR_2$ = 0.0399 |
| Largest diff. peak/hole [e Å$^{-3}$] | 1.22/-1.06 |
| **Refinement results for the MM** | |
| Data/restraints/parameters | 16855/0/530 |
| Goodness-of-fit on $F^2$ | 1.005 |
| Final R indexes [I>=2σ (I)] | $R_1$ = 0.0149 |
| Final R indexes [all data] | $R_1$ = 0.0165, $wR_2$ = 0.0292 |
| Largest diff. peak/hole / e Å$^{-3}$ | 0.55/-0.54 |

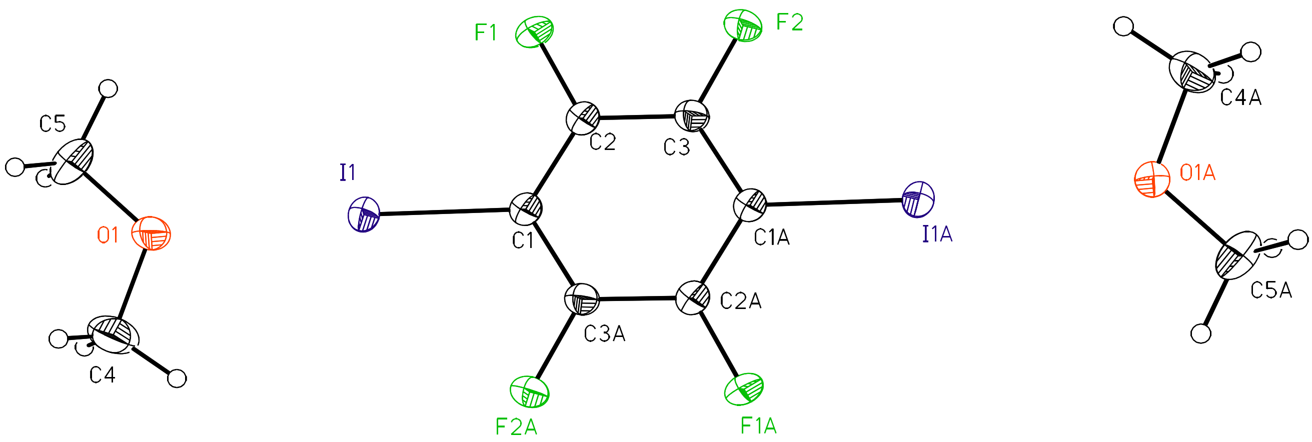


**Figure S8.** Displacement ellipsoid plot of the structure of the halogen bond adduct **5** in the crystal with ellipsoids drawn at 50% probability. Numbering of hydrogen atoms omitted for clarity. Selected distances [Å] and [°]: I1–O1 2.846(3), C1–I1 2.082(3), C1–I1–O1 175.85(12). CCDC deposition number 2334581.

**Table S5.** Crystallographic data and structural refinements for **5**.

| | **5** |
|---|---|
| Empirical formula | $C_{10}H_{12}F_4I_2O_2$ |
| Formula weight [g·mol$^{-1}$] | 494.00 |
| Temperature [K] | 100.00 |
| Crystal system | triclinic |
| Space group | $P\bar{1}$ |
| a [Å] | 6.9565(3) |
| b [Å] | 7.0420(3) |
| c [Å] | 9.2939(4) |
| $\alpha$ [°] | 103.9440(17) |
| $\beta$ [°] | 98.4014(19) |
| $\gamma$ [°] | 116.9543(16) |
| Volume [Å$^3$] | 376.32(3) |
| Z | 1 |
| $\rho_{calc}$ [g/cm$^3$] | 2.180 |
| μ [mm$^{-1}$] | 4.213 |
| F(000) | 230.0 |
| Crystal size [mm$^3$] | 0.234 × 0.167 × 0.135 |
| Radiation | MoK$\alpha$ ($\lambda$ = 0.71073) |
| 2Θ range for data collection [°] | 6.84 to 72.868 |
| Index ranges | $-11 \leq h \leq 11$<br>$-11 \leq k \leq 11$<br>$-15 \leq l \leq 15$ |
| Reflections collected | 20795 |
| Independent reflections | 3669<br>[$R_{int}$ = 0.0446, $R_{sigma}$ = 0.0313] |
| Data/restraints/parameters | 3669/0/106 |
| Goodness-of-fit on $F^2$ | 1.070 |
| Final R indexes [I>=2σ (I)] | $R_1$ = 0.0397, $wR_2$ = 0.0959 |
| Final R indexes (all data) | $R_1$ = 0.0471, $wR_2$ = 0.1010 |
| Largest diff. peak/hole [e Å$^{-3}$] | 1.89/-2.6 |

## 2 CSD Database Search

Different CSD Database Searches were conducted.[2] The search parameters, results and selected histograms are presented.

a) **search query: O–I distance < 3.2 Å; 160° < O–I–C angle < 180°; monosubstituted iodine**

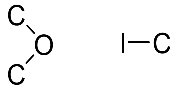

112 structures were found. This number displays the halogen bonds between ether oxygen and iodine found in literature.

b) **search query: O–I distance < 3.2 Å; 160° < O–I–C angle < 180°; no disorder; monosubstituted iodine**

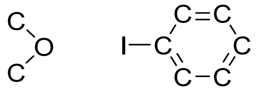

49 structures were found.

C–I bond distance also depends on substituents an aryliodine compound. Fluorinated compounds show similar bond distances to compounds **1-5** (Figure S9). The wide flexibility of O···I distance is also reflected in literature known structures (Figure S10).

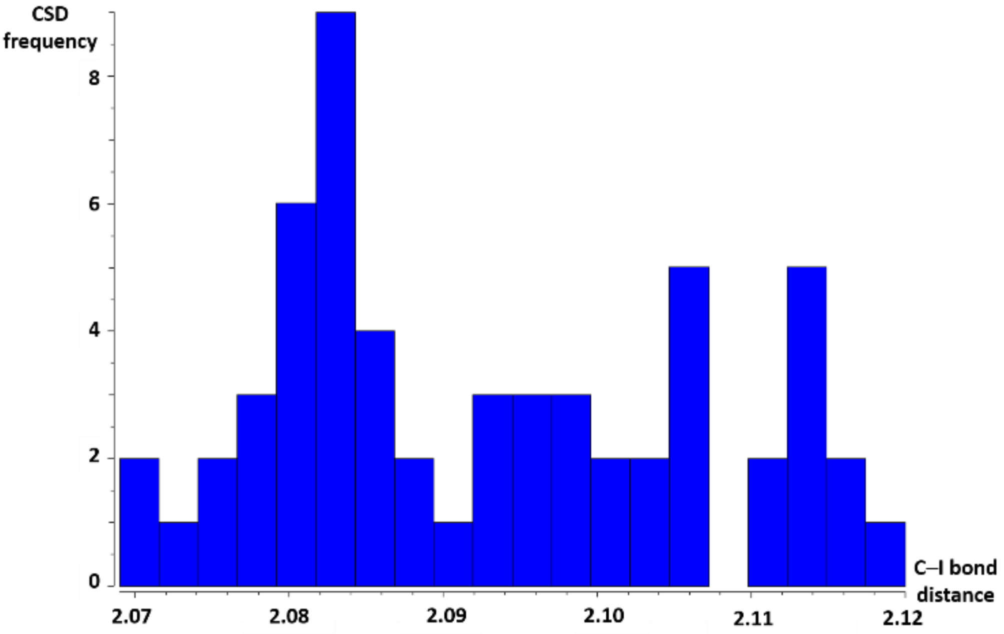


**Figure S9.** Histogram of C–I bond distances found in CSD database for ether-oxygen to aryl-iodine; O–I distance < 3.2 Å; 160° < O–I–C angle < 180°; no disorder.

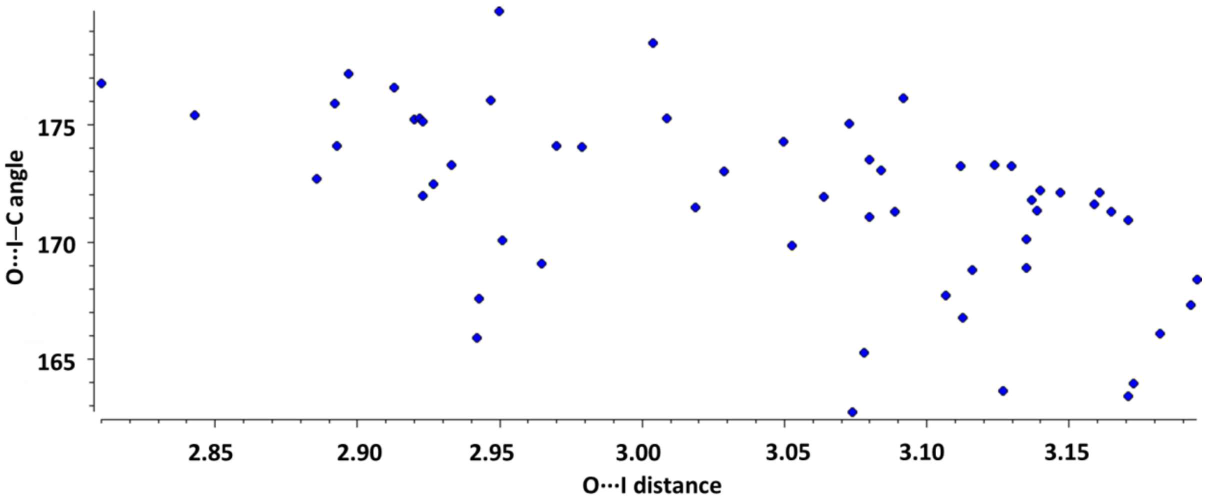


**Figure S10.** Plot of O···I–C angle against O···I distance found in CSD database for ether-oxygen to aryl-iodine; O–I distance < 3.2 Å; 160° < O–I–C angle < 180°; no disorder.

**c) search query: no disorder; 3D coordinates determined**

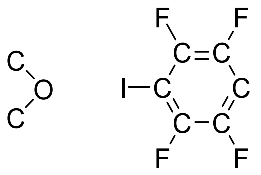

26 structures were found and can be classified by the following ether motifs:

- Arly-O-Me: IWOMUE, IWOPAN, JEGKOY, MENHEW, PEMKAX, PIRFOP, QAVTIU, SEDFUF, SEDFUF01, SEDGOA, SEDGOA01, TONMIT, VAZMIV (13)
- Morpholine-derived: AJOFEN, AJOHOZ, AJOHUF, BENJOX, BENKAK, NERDOH, QUQXOQ (7)
- 1,4-dioxane: DIVDAO, XAXPUL (2)
- Aryl-O-Aryl: OLOQEO, WUKLAR (2)
- Aryl-O-Alkyl: DURHEG, RUYJEB (2)

No structures with dialkyl ethers were found.

**d) Search query: 2.5 Å < O–I distance < 3.5 Å**

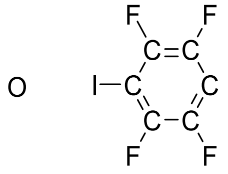

218 structures were found.

Figure 11 shows the O–I bond distance. Pyridine oxide and carboxylate groups are responsible for short distances, these hits are marked orange in Figure 11.

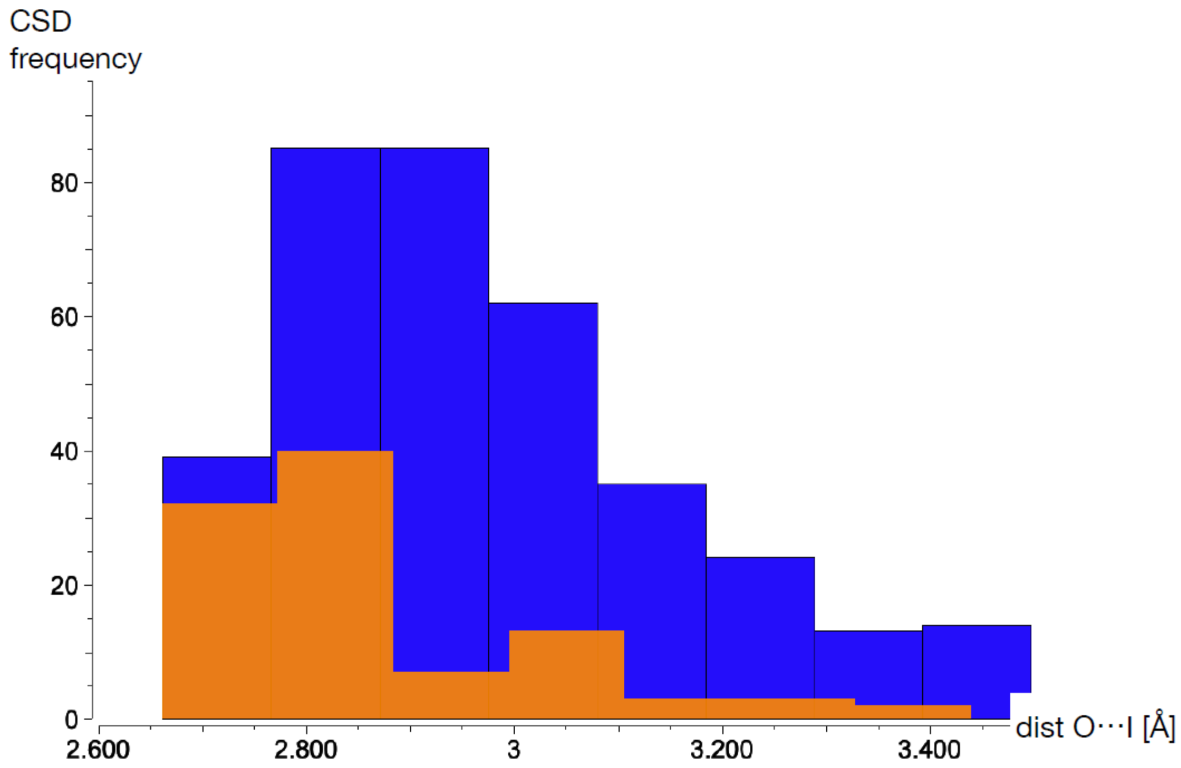


**Figure S11.** Histogram of O–I distances for all close contacts between an oxygen centre and tetrafluoroiodobenzenes. Pyridine oxide and carboxylate group containing structures are marked orange.

**e) search query:**

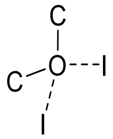


5 structures (KELFEO, LOMGUQ, RIKCET, XAHSEH, ZIYRII) were found, whereas none of them presents short O–I distances (<3.2 Å).

# 3 Experimental Electron Density Study

## 3.1 Statistics of the electron density study

Results for the electron density study of XB adduct **4** are given below.

**Table S6.** Resolution and completeness statistics (cumulative and Friedel pairs averaged).

| Theta | sin(th)/Lambda | Complete | Expected | Measured | Missing |
|---|---|---|---|---|---|
| 20.82 | 0.500 | 1.000 | 1236 | 1236 | 0 |
| 23.01 | 0.550 | 1.000 | 1633 | 1633 | 0 |
| 25.24 | 0.600 | 1.000 | 2145 | 2145 | 0 |
| 27.51 | 0.650 | 1.000 | 2714 | 2714 | 0 |
| 29.84 | 0.700 | 1.000 | 3397 | 3397 | 0 |
| 32.21 | 0.750 | 1.000 | 4184 | 4184 | 0 |
| 34.65 | 0.800 | 1.000 | 5065 | 5065 | 0 |
| 37.17 | 0.850 | 1.000 | 6081 | 6081 | 0 |
| 39.77 | 0.900 | 1.000 | 7214 | 7214 | 0 |
| 42.47 | 0.950 | 1.000 | 8474 | 8474 | 0 |
| 45.29 | 1.000 | 1.000 | 9897 | 9897 | 0 |
| 48.27 | 1.050 | 1.000 | 11466 | 11466 | 0 |
| 51.43 | 1.100 | 1.000 | 13154 | 13154 | 0 |
| 54.82 | 1.150 | 1.000 | 15051 | 15051 | 0 |
| 58.50 | 1.200 | 1.000 | 17076 | 17076 | 0 |

Note: The Reported Completeness refers to the Actual H,K,L Index Range

**Table S7.** R-value statistics as a function of resolution (in resolution shells).

| Theta | sin(Th)/L | # | R1 | wR2 | S | Rs | av(I/SigW) | av(I) | av(SigW) |
|---|---|---|---|---|---|---|---|---|---|
| 12.38 | 0.302 | 272 | 0.012 | 0.038 | 2.586 | 0.004 | 65.15 | 13339.23 | 168.86 |
| 15.68 | 0.380 | 277 | 0.011 | 0.034 | 2.151 | 0.004 | 59.47 | 7288.12 | 96.57 |
| 18.02 | 0.435 | 266 | 0.013 | 0.034 | 2.007 | 0.005 | 53.26 | 5161.27 | 71.25 |
| 19.90 | 0.479 | 270 | 0.014 | 0.034 | 1.904 | 0.005 | 49.82 | 4019.99 | 57.76 |
| 21.51 | 0.516 | 275 | 0.012 | 0.028 | 1.521 | 0.006 | 50.33 | 3281.30 | 49.60 |
| 22.94 | 0.548 | 262 | 0.013 | 0.027 | 1.413 | 0.006 | 46.56 | 2770.96 | 43.78 |
| 24.22 | 0.577 | 287 | 0.015 | 0.029 | 1.381 | 0.007 | 40.23 | 2358.73 | 38.53 |
| 25.40 | 0.603 | 270 | 0.012 | 0.026 | 1.231 | 0.007 | 42.15 | 1999.55 | 34.31 |
| 26.49 | 0.628 | 265 | 0.014 | 0.028 | 1.202 | 0.009 | 36.34 | 1418.08 | 26.92 |
| 27.52 | 0.650 | 270 | 0.015 | 0.028 | 1.208 | 0.009 | 36.70 | 1514.34 | 28.20 |
| 28.49 | 0.671 | 280 | 0.013 | 0.026 | 1.095 | 0.009 | 36.67 | 1324.51 | 25.89 |
| 29.41 | 0.691 | 270 | 0.017 | 0.034 | 1.399 | 0.010 | 35.26 | 1165.46 | 23.42 |
| 30.28 | 0.709 | 259 | 0.017 | 0.033 | 1.248 | 0.010 | 32.98 | 956.03 | 20.33 |
| 31.12 | 0.727 | 280 | 0.016 | 0.034 | 1.330 | 0.009 | 33.72 | 951.01 | 19.91 |
| 31.93 | 0.744 | 292 | 0.018 | 0.038 | 1.435 | 0.009 | 32.11 | 871.96 | 18.44 |
| 32.71 | 0.760 | 259 | 0.018 | 0.041 | 1.445 | 0.010 | 30.88 | 645.98 | 15.17 |
| 33.46 | 0.776 | 258 | 0.017 | 0.040 | 1.444 | 0.009 | 31.52 | 680.85 | 15.50 |
| 34.20 | 0.791 | 276 | 0.021 | 0.048 | 1.609 | 0.009 | 28.66 | 626.24 | 14.13 |
| 34.91 | 0.805 | 284 | 0.018 | 0.040 | 1.387 | 0.009 | 29.84 | 635.31 | 14.46 |
| 58.50 | 1.200 | 11904 | 0.026 | 0.053 | 1.005 | 0.021 | 14.66 | 163.81 | 6.55 |

R(sig) = sum(sig(I)) / sum(I) = 0.0077

## 3.2 Refinement details

Refinement was conducted with all intensity data $I > 0$. The final refinements on $F^2$ comprised multipoles up to hexadecapoles for non-H atoms and up to bond directed dipoles for the H atoms. Contraction parameters for non-H atoms and $\varkappa'$ for I and F were refined freely; $\varkappa'$ was fixed to 1.0 for O and C, to 1.2 for H atoms. Contraction parameters and monopole population coefficients have been compiled in Table S12. The positional parameters of H atoms were fixed to distance C–H 1.09 Å .

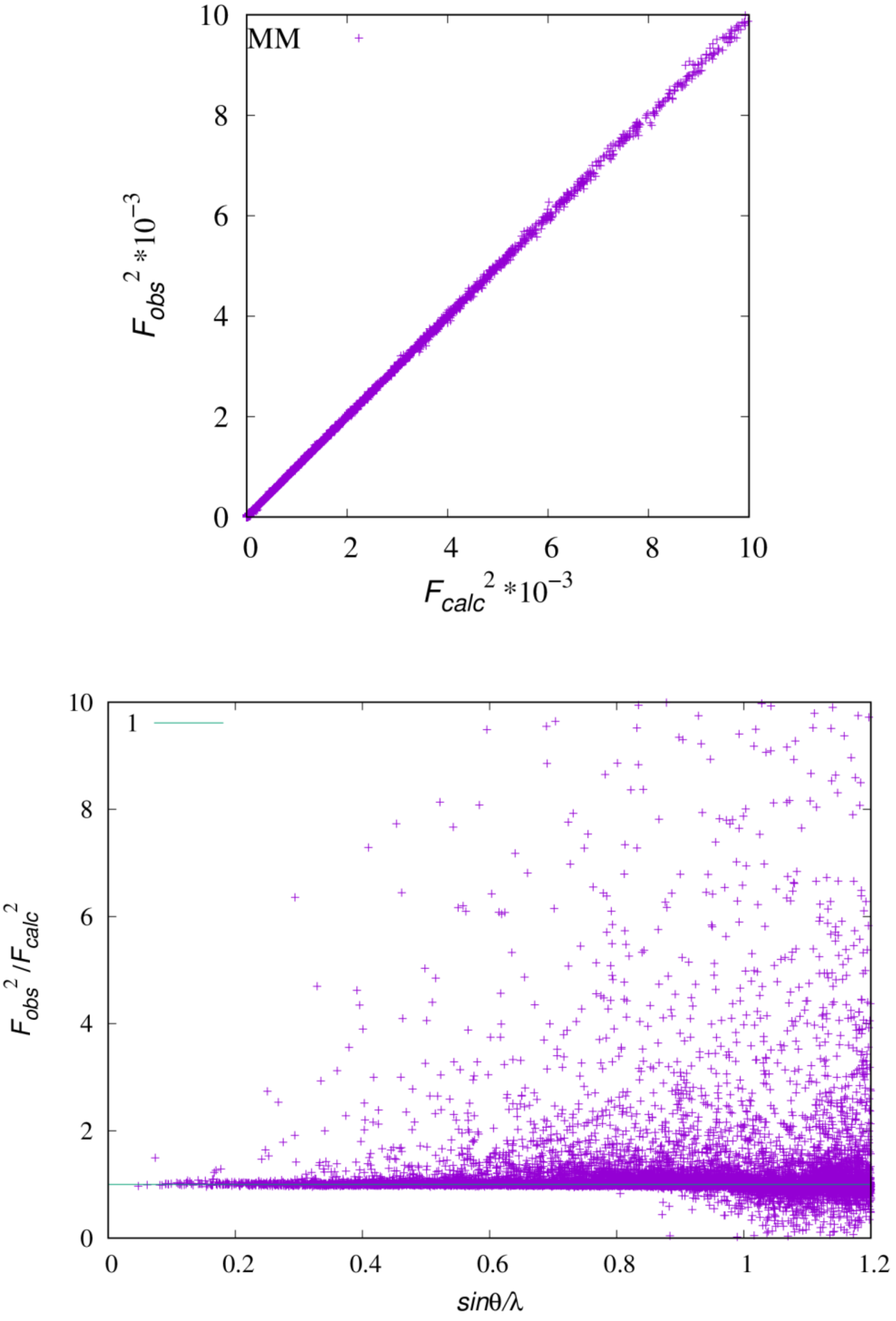


**Figure S12.** Scatterplot for X-ray refinement result (MM).

**Table S8.** Final Coordinates of the non-hydrogen atoms in the XB adduct.

| | | | |
|---|---|---|---|
| I(1) | 0.797286(16) | 0.562746(4) | 0.740550(13) |
| I(2) | 0.188648(14) | 0.284252(4) | 0.696885(11) |
| F(1) | 0.38232(18) | 0.52580(4) | 0.86200(14) |
| F(2) | 0.15453(17) | 0.41934(5) | 0.85020(14) |
| F(3) | 0.58555(19) | 0.32364(5) | 0.55427(14) |
| F(4) | 0.81356(19) | 0.43035(6) | 0.56509(14) |
| O(1) | -0.08194(11) | 0.17296(3) | 0.67259(8) |
| C(1) | 0.60757(10) | 0.48121(3) | 0.71603(8) |
| C(2) | 0.43466(11) | 0.47656(3) | 0.78510(8) |
| C(3) | 0.31550(10) | 0.42111(3) | 0.77897(8) |
| C(4) | 0.36440(10) | 0.36797(3) | 0.70291(7) |
| C(5) | 0.53427(11) | 0.37282(3) | 0.63141(7) |
| C(6) | 0.65357(11) | 0.42846(3) | 0.63728(8) |
| C(7) | -0.19749(17) | 0.16515(4) | 0.51893(9) |
| C(8) | -0.22424(15) | 0.17752(4) | 0.77129(10) |

**Table S9.** Anisotropic displacement parameters for the XB adduct.

| Uij | values U11 | U22 | U33 | U12 | U13 | U23 |
|---|---|---|---|---|---|---|
| I(1) | 0.01692(3) | 0.01557(3) | 0.02219(4) | -0.00245(3) | 0.00242(3) | 0.00119(3) |
| I(2) | 0.01604(3) | 0.01552(3) | 0.01985(3) | -0.00214(2) | 0.00436(3) | 0.00025(3) |
| F(1) | 0.0242(3) | 0.0171(3) | 0.0281(3) | 0.0018(2) | 0.0094(3) | -0.0040(3) |
| F(2) | 0.0221(3) | 0.0223(3) | 0.0272(3) | -0.0008(3) | 0.0135(3) | -0.0017(3) |
| F(3) | 0.0258(3) | 0.0203(3) | 0.0270(3) | -0.0028(3) | 0.0133(3) | -0.0071(3) |
| F(4) | 0.0227(3) | 0.0250(3) | 0.0280(4) | -0.0048(3) | 0.0140(3) | -0.0035(3) |
| O(1) | 0.01686(19) | 0.0177(2) | 0.0190(2) | -0.00067(16) | 0.00522(17) | -0.00140(17) |
| C(1) | 0.01554(18) | 0.01512(18) | 0.0180(2) | -0.00089(15) | 0.00364(16) | -0.00001(15) |
| C(2) | 0.01644(19) | 0.01459(18) | 0.0189(2) | 0.00059(15) | 0.00484(16) | -0.00082(15) |
| C(3) | 0.01580(18) | 0.01575(18) | 0.01800(19) | -0.00008(15) | 0.00589(16) | -0.00025(16) |
| C(4) | 0.01530(17) | 0.01481(17) | 0.01631(18) | -0.00082(14) | 0.00456(15) | -0.00020(14) |
| C(5) | 0.01685(19) | 0.01586(18) | 0.01728(19) | -0.00135(15) | 0.00593(16) | -0.00170(15) |
| C(6) | 0.01643(19) | 0.0175(2) | 0.0182(2) | -0.00198(16) | 0.00619(16) | -0.00112(16) |
| C(7) | 0.0306(3) | 0.0202(3) | 0.0183(2) | -0.0024(2) | 0.0043(2) | 0.00020(19) |

**Table S10.** Bond distances for the XB adduct.

| | | | |
|---|---|---|---|
| I(1)$^{i}$ …O(1) | 2.9326(8) | I(2) …O(1) | 2.8850(8) |
| I(1) - C(1) | 2.0801(6) | I(2) - C(4) | 2.0823(6) |
| F(1) - C(2) | 1.3386(11) | F(2) - C(3) | 1.3345(10) |
| F(3) - C(5) | 1.3364(11) | F(4) - C(6) | 1.3350(11) |
| O(1) - C(7) | 1.4224(11) | O(1) - C(8) | 1.4212(10) |
| C(1) - C(2) | 1.3918(9) | C(1) - C(6) | 1.3925(9) |
| C(2) - C(3) | 1.3866(9) | C(3) - C(4) | 1.3916(9) |
| C(4) - C(5) | 1.3893(9) | C(5) - C(6) | 1.3906(9) |

i = 1-x, -0.5+y, 1.5-z

**Table S11.** Bond angles for the XB adduct.

| | | | |
|---|---|---|---|
| I(1)i…O(1) …I(2) | 108.06(2) | C(7) - O(1) - C(8) | 112.00(7) |
| I(1) - C(1) - C(2) | 120.48(5) | I(1) - C(1) - C(6) | 121.97(5) |
| C(2) - C(1) - C(6) | 117.48(6) | F(1) - C(2) - C(1) | 120.18(7) |
| F(1) - C(2) - C(3) | 118.38(7) | C(1) - C(2) - C(3) | 121.44(6) |
| F(2) - C(3) - C(2) | 118.46(7) | F(2) - C(3) - C(4) | 120.46(7) |
| C(2) - C(3) - C(4) | 121.06(6) | I(2) - C(4) - C(3) | 121.02(5) |
| I(2) - C(4) - C(5) | 121.33(5) | C(3) - C(4) - C(5) | 117.65(6) |
| F(3) - C(5) - C(4) | 120.18(7) | F(3) - C(5) - C(6) | 118.51(7) |

**Table S12.** Monopole populations, radial parameters and net atomic charges for the XB adduct.

| Atom | $P_{val}$ | Kappa | Kappa' | Atomic charge |
|---|---|---|---|---|
| I(1) | 7.02(6) | 1.008 | 3.686 | -0.02(6) |
| I(2) | 7.08(6) | 1.008 | 3.686 | -0.08(6) |
| F(1) | 7.07(3) | 0.992 | 1.098 | -0.07(3) |
| F(2) | 7.11(3) | 0.992 | 1.098 | -0.11(3) |
| F(3) | 7.13(3) | 0.992 | 1.098 | -0.13(3) |
| F(4) | 7.11(3) | 0.992 | 1.098 | -0.11(3) |
| O(1) | 6.18(3) | 0.987 | 1.000 | -0.18(3) |

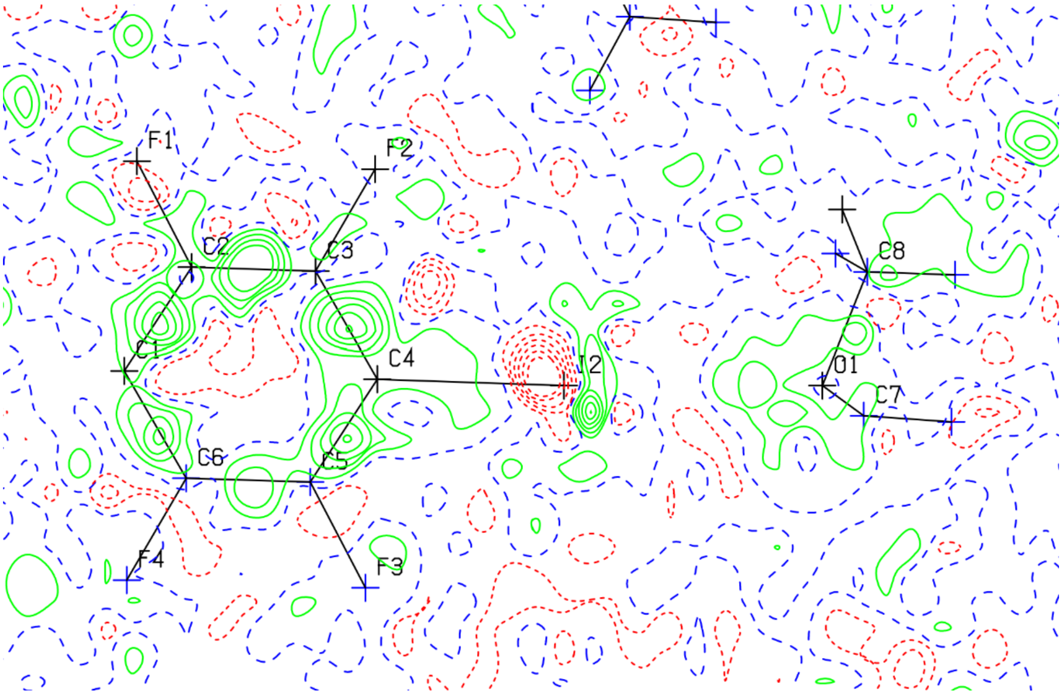


**Figure S13.** Difference Fourier maps for the IAM refinement in the plane subtended by I2, O1 and C3. Contour lines have been drawn at 0.1 eA$^{-3}$; green contours correspond to positive, red to negative electron densities.

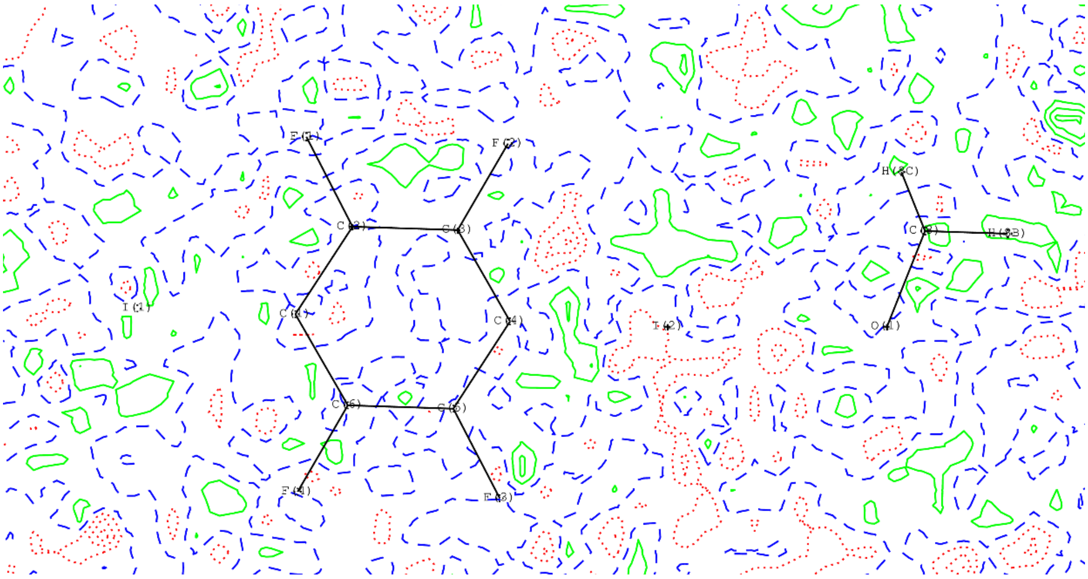

**Figure S14.** Difference Fourier maps for the MM refinement in the plane subtended by I2, O1 and C3. Contour lines have been drawn at 0.1 eA$^{-3}$; green contours correspond to positive, red to negative electron densities.

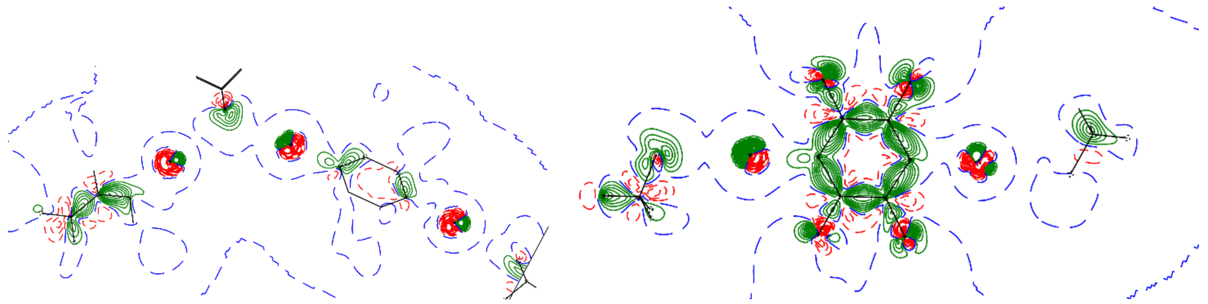

**Figure S15.** Deformation density map for the XB adduct after the IAM refinement. The contour interval is 0.10 0.10 eÅ$^{-3}$, green lines indicate positive, red lines negative and blue lines zero contours.

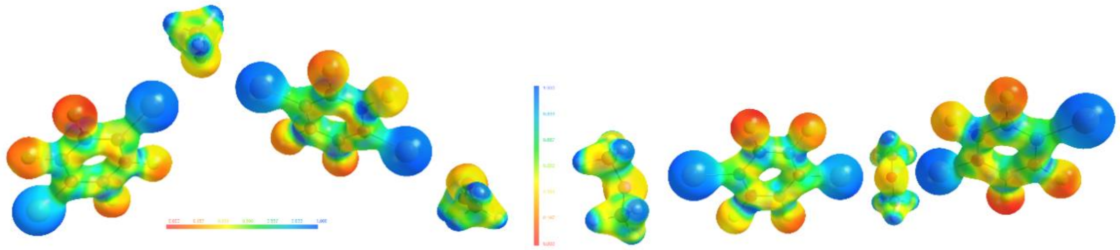

**Figure S16.** Electrostatic potential mapped on an isosurface of electron density ($\rho$ = 0.5 e·Å$^{-3}$) of the XB adduct (program *MoleCoolQt*).

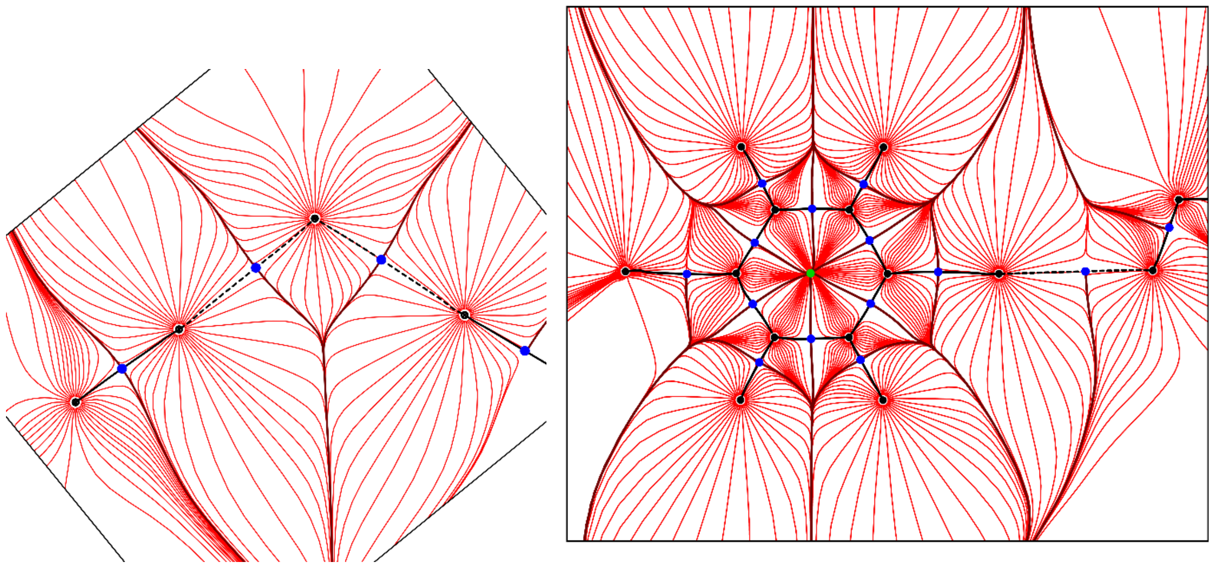

**Figure S17.** Trajectory plots for Gradient of Electron Density.

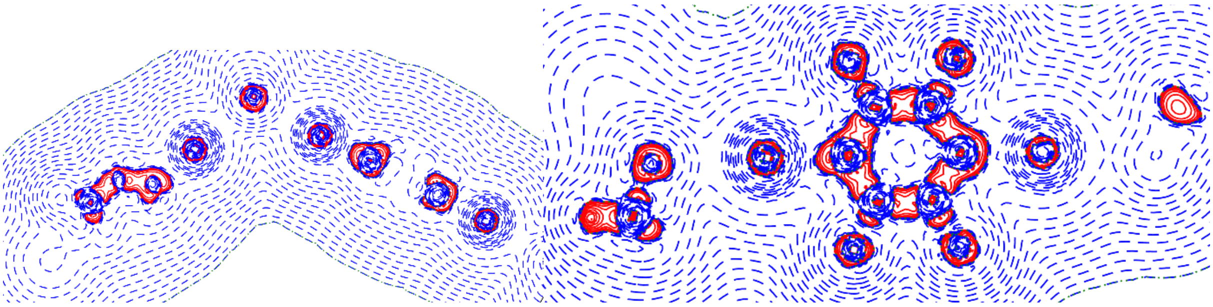

**Figure S18.** Laplacian of the electron density of the XB adduct, with positive values in blue, negative values in red and contours at $\pm 2^n \cdot 10^{-3}$ e·Å$^{-5}$.

**Table S13.** Properties of the electron density in the bcps of the halogen bond XB adduct. $d_1$ ($d_2$) is the distance from the first (second) atom to (3,-1) critical point, $R_{ij} = d_1 + d_2$, $\rho$ is the electron density, $\nabla^2 \rho$ is the Laplacian of the electron density. $G(a.u.)$ is the kinetic energy density $G / \rho(a.u..)$ the ration between kinetic energy and electron density, $V(a.u.)$ the potential energy density and $E(a.u.)$ the total energy density in the bond critical point.

| Bond | $R_{ij}$(Å) | $d_1$(Å) | $d_2$(Å) | $\rho(e{\cdot}Å^{-3})$ | $\nabla^2\rho(e{\cdot}Å^{-5})$ | $G(a.u.)$ | $\frac{G}{\rho}(a.u.)$ | $V(a.u.)$ | $E(a.u.)$ |
|---|---|---|---|---|---|---|---|---|---|
| O1···I2 | 2.8850 | 1.2651 | 1.6199 | 0.160(4) | 1.789(4) | 0.0180 | 0.76 | -0.0174 | 0.0006 |
| O1$^i$···I1 | 2.9325 | 1.2857 | 1.6468 | 0.146(4) | 1.604(4) | 0.0159 | 0.74 | -0.0152 | 0.0007 |
| C1—I1 | 2.0823 | 0.9236 | 1.1587 | 0.74(4) | 4.10(9) | | | | |
| C4—I2 | 2.0832 | 0.9465 | 1.1367 | 0.84(4) | 2.09(8) | | | | |
| F1—C2 | 1.3388 | 0.7889 | 0.5499 | 2.17(8) | -28.0(4) | | | | |
| F2—C3 | 1.3350 | 0.7854 | 0.5496 | 2.08(8) | -17.5(4) | | | | |
| F3—C5 | 1.3367 | 0.8635 | 0.4732 | 2.04(8) | -26.0(5) | | | | |
| F4—C6 | 1.3364 | 0.7859 | 0.5505 | 2.11(8) | -21.0(4) | | | | |
| O1—C7 | 1.4229 | 0.8261 | 0.5968 | 1.9(2) | -15.5(4) | | | | |
| O1—C8 | 1.4246 | 0.8590 | 0.5656 | 1.8(2) | -19.2(5) | | | | |
| C1—C2 | 1.3923 | 0.6692 | 0.7232 | 2.16(7) | -18.6(3) | | | | |
| C1—C6 | 1.3936 | 0.6501 | 0.7434 | 2.20(7) | -20.8(3) | | | | |
| C2—C3 | 1.3871 | 0.6927 | 0.6944 | 2.24(7) | -22.6(3) | | | | |
| C3—C4 | 1.3931 | 0.6834 | 0.7098 | 2.19(7) | -19.8(3) | | | | |
| C4—C5 | 1.3901 | 0.6422 | 0.7479 | 2.2(2) | -18.6(5) | | | | |
| C5—C6 | 1.3915 | 0.7107 | 0.6808 | 2.2(2) | -20.3(5) | | | | |

$i = 1 - x, 0.5 + y, 1.5 - z$

### 3.3 Residual electron density distribution

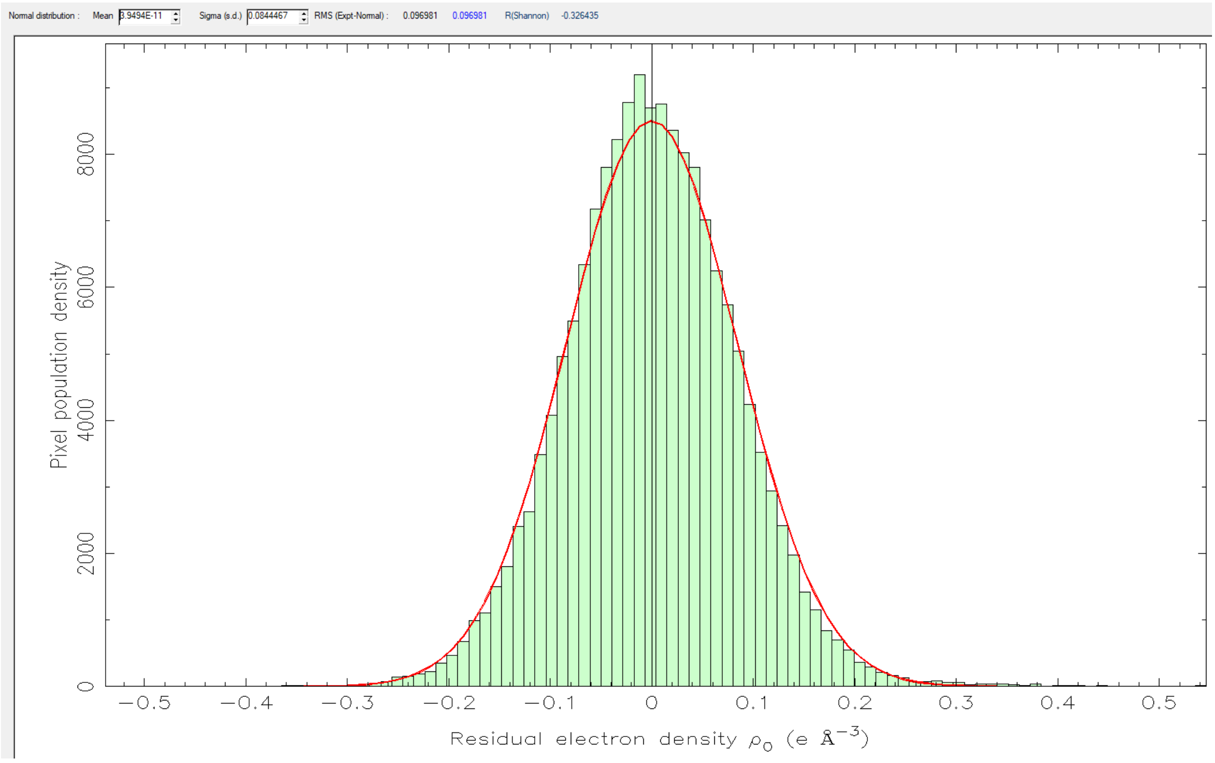


**Figure S19.** Probability distribution histogram of residual electron density of XB adduct.

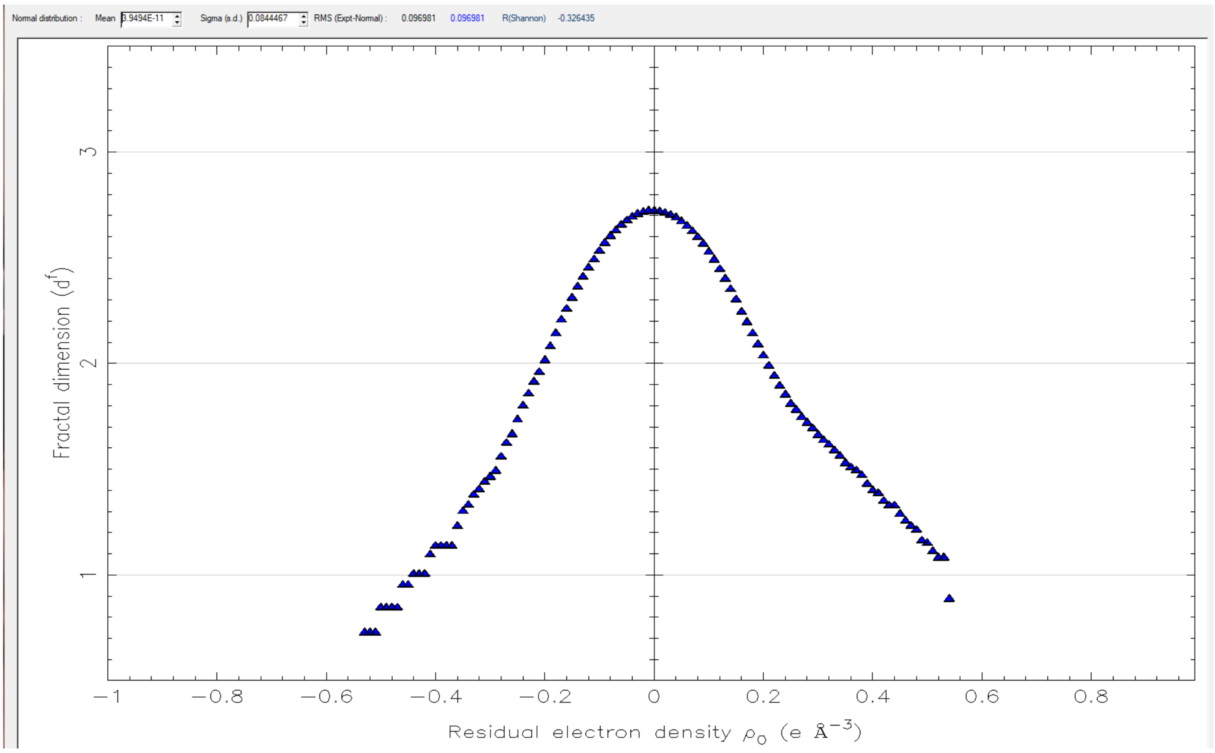


**Figure S20.** Fractal dimension plot of residual electron density of XB adduct.

## References


[1] F. Otte, J. Kleinheider, B. Grabe, W. Hiller, F. Busse, R. Wang, N. M. Kreienborg, C. Merten, U. Englert, C. Strohmann, *ACS Omega* **2023**, *8*, 21531–21539.

[2] C. R. Groom, I. J. Bruno, M. P. Lightfoot, S. C. Ward, *Acta Crystallogr*. **2016**, *B72*, 171−179; version 5.45.


## Author Contributions

A.S.: writing of original draft, writing of SI, formal analysis

A.K.: crystallization of compound **1**, **2**, **4**, **5**; data curation, formal analysis, investigation

J.K.: NMR titration experiments

T.B.: crystallization of compound **3**

R.W.: preparation of electron density study via MM refinement, writing of SI.

U.E.: preparation of electron density study via IAM refinement, funding acquisition, validation

C.S.: funding acquisition, investigation, project administration, validation
